# Reusability-targeted enrichment of sea ice core data

**DOI:** 10.1038/s41597-025-04665-x

**Published:** 2025-03-20

**Authors:** Anna Simson, Anil Yildiz, Julia Kowalski

**Affiliations:** Methods for Model-based Development in Computational Engineering, RWTH Aachen University, Aachen, Germany

**Keywords:** Cryospheric science, Databases

## Abstract

The Reusability-targeted Enriched Sea Ice Core Database (RESICE) combines data and metadata from 287 sea ice cores. The database enables reuse scenarios such as the validation of physics-based models and the training of data-driven algorithms. RESICE is enriched in two ways. First, RESICE combines data and metadata originating from 138 sources including 107 data sets from the repositories Zenodo, Australian Antarctic Data Center and Pangaea. Second, RESICE contains additional automatically generated metadata tailored to specific reuse scenarios. RESICE is checked for plausibility and consistency, and it allows transparent retracing of each data point to its source. RESICE is accessible via Zenodo and the MOSAiC webODV, and it is extendable through the pyresice Python package. In addition to describing RESICE, we formalize the reuse perspective of an agnostic reuser, uninvolved in data acquisition, and we discuss the process of the cross-source and -repository combination of the database. Despite sources adhering to FAIR, this process is challenging and time-intensive due to the heterogeneity of the sources and their mismatch with reuse requirements.

## Background & Summary

Scientists from all over the world acquire data from sea ice cores drilled in Earth’s polar regions^1–4^. An increasing number of the data sets acquired are becoming publicly available allowing other researchers to use the data and address new research questions. The use of existing data for purposes different than that of data collection is called *reuse*^5–7^. While it is argued that a distinction between the terms *use* and *reuse* may not be necessary^8^, we constrain *reuse* in this article to the secondary usage of published material by another person than the creator.

We refer to the problems addressed within this secondary usage as *reuse scenarios*. Reuse scenarios can include a wide variety of tasks such as re-plotting, validating models, training data-driven models or developing digital twins. In the realm of sea ice core data, exemplary reuse scenarios are the validation of physics-based process models for sea ice evolution and the data-driven classification of qualitative sea ice characteristics. Reuse scenarios often come from domains other than the data origin and are performed by people not involved in the data collection. These reusers are data agnostic, and their idea of the data is driven by the requirements of reuse scenarios without knowing about actual data availabilities. Data reusers may request data and metadata that do not exist or are unavailable in the required quality from existing data sets.

The FAIR principles^9^ should facilitate these agnostic reuses as they have been formulated to ensure findability, accessibility, interoperability, and ultimately reusability of data. If data is published by FAIR-compliant repositories, agnostic reusers could assume sea ice core data is easily integrable into their scenarios. In reality, while this may hold for a single sea ice core data set, the combined cross-source and -repository reuse of multiple sea ice core data sets is not possible without significant time investment. In this article, we focus on agnostic reusers. However, data collectors also find it challenging to combine their own data.

**Fig. 1:**
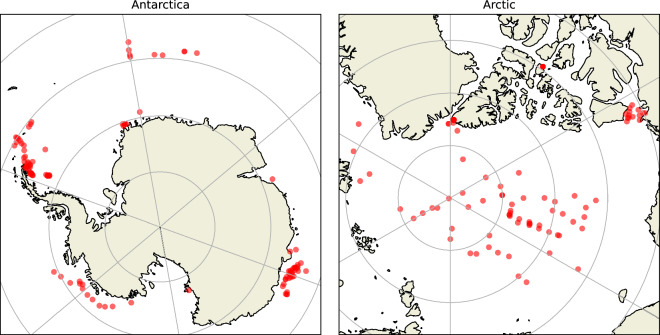
Locations of all sea ice cores incorporated in RESICE at the time of submission. Color intensity indicates density of available sea ice cores. The map is generated with Cartopy^76^.

Sea ice core data sets typically do not align with reuse needs, as they are structured to best reflect data collection and not to best serve reuse purposes. The combination of various data sets into a homogeneous form is impeded by the heterogeneity within and between data sets. The data sets vary in quality, descriptiveness, content, label names, units, and formats. Relevant data and metadata are not available from data sets but from context providing sources, such as articles or expedition reports. The data and metadata of the same sea ice core can be distributed across several data sets, and it can be redundant on different repositories. The Reusability-targeted Enriched Sea Ice Core Database (RESICE) addresses these challenges and combines data and metadata of 287 sea ice cores from locations indicated in Fig. 1. All data points in RESICE can be traced back to their original sources. The tabular database is accessible via Zenodo^10,11^ and the MOSAiC webODV (https://mvre.webodv.cloud.awi.de/service/Extra>Sea_Ice>RESICE), and it is extendable by other researchers through the pyresice Python package^12^ (https://git.rwth-aachen.de/mbd/pyresice).

RESICE enrichment increases the reusability of all combined data sets and is achieved in two ways. First, RESICE combines data and metadata originating from 138 sources, including 107 data sets from the repositories Zenodo, Australian Antarctic Data Center (AADC) and Pangaea, 23 articles and reports, and 8 instrument manuals. Second, RESICE contains additional automatically generated metadata tailored to the requirements of the specific reuse scenarios and systematically derived based on Python routines programmed by the data reuser. Automatic metadata enrichment is necessary because not all required metadata is available or is not available in the required form or quality. The mean distance of measurement data along a core may be required, but it may not be explicitly provided. However, the mean distance can be inferred from the depths of the measurements provided by the existing data. Furthermore, metadata can be automatically enriched by using existing metadata. For example, by using a shapefile that provides polygons with sea names according to standard naming conventions, the coordinates of a core can be used to find the corresponding sea name.

This article serves two purposes. First, we present the reusability-targeted approach followed to curate and enrich a combined database across sources and repositories. Second, we describe the database RESICE in order to facilitate its reuse by others in further scenarios.

The reusability-targeted approach can be summarized as follows:

- **Step 1** Formalization of the reuse perspective with
  - reuse scenarios
  - reuse scope, i.e., the required data and metadata, combined referred to as elements
- **Step 2** Assembly of reuse scope relevant data and metadata by
  - searching for sources
  - matching elements of the reuse scope that are available from sources
- **Step 3** Plausibility checks of the sources
- **Step 4** Technical combination of the sources
- **Step 5** Automatic metadata enrichment of unavailable reuse scope elements

Intuitively, one assumes all information contained in the reuse scope is available from sources. Accordingly, agnostic data reusers would dedicate most time for Step 2 (a) assuming Step 3 and Step 4 would be easy. In reality, even data considered highly FAIR by the assessment tool *F-UJI*^13^ are distributed across sources and are difficult to combine. Therefore, significant time is necessary for Step 2 (b), namely matching reuse scope elements with source availabilities, and for Steps 3 and 4. After combining all data and metadata available from existing sources in Step 4, missing reuse scope elements are automatically enriched on the basis of Python routines developed by the data reuser in Step 5. The following methods section describes the combination of the tabular database RESICE aligned with the steps of the reusability-targeted approach. It is followed by a description of the provided data records, the technical validation of RESICE and notes on the usage of RESICE.

## Methods

The structure of this section follows the five steps of the reusability-targeted approach, starting with the formalization of the reuse perspective. This is demonstrated at the example of two typical reuse scenarios that motivate the compilation of RESICE.

### Step 1 (a): Reuse scenarios

Reuse of data is performed in a context and for a task. The clear description of the reuse purpose and the clear definition of the reuse requirements should be the first step and preceed the search for sources. Reuse scenarios vary in purposes and require individual inputs in form of data and metadata. Scenarios could be A the validation of a sea ice evolution model or B the training of a classification algorithm to detect qualitative features of sea ice. The validation of a heat flux simulation, for instance, requires temperature measurement data as input. Depending on the scenario, these inputs can be further specified by constraints, e.g., minimum mean distance of measurements. Further examples for constraints are specific units, formats, value limits, maximum allowed measurement errors, compliance with specific naming standards and others. Inputs have to comply with the constraints to be suitable for the scenario. This is best exemplified based on two specific reuse scenarios defined in the following. The inputs and constraints of both scenarios are listed in Table 1, which constitutes the formalization of the reuse perspective.

**Table 1:** List of inputs and constraints for Reuse Scenarios A and B.

Elements	Reuse Scenario A		Reuse Scenario B	
	Input	Constraints	Input	Constraints
Date			*✓*	Format: YYYY-MM-DD
Coordinates			*✓*	Format: decimal degrees
Water body			*✓*	with SeaVoX class names
Salinity sea ice (vertical profile)	*✓*	Ratio (salinity): ppt Unit (depth): m Mean distance: below 0.2 m Measurement error: below 5%		
Solid fraction sea ice (vertical profile)	*✓*	Unit (solid fraction): - Unit (depth): m Mean distance: below 0.2 m Measurement error: below 5%		
Temperature sea ice (vertical profile)	*✓*	Unit (temperature): K Unit (depth): m Mean distance: below 0.2 m Measurement error: below 5%		
Temperature air	*✓*	Unit: K		
Salinity sea water	*✓*	Ratio: ppt		
Temperature sea water	*✓*	Unit: K		
Thickness snow	*✓*	Unit: m		
Thickness sea ice	*✓*	Unit: m Value: below 1 m	*✓*	Unit: m
Measurement errors	*✓*	Ratio: %		
Standard deviations of repeated measurements	*✓*			
Mean salinity sea ice			*✓*	Ratio: ppt
Mean temperature sea ice			*✓*	Unit: K
Form sea ice			*✓*	with SIN class names
Development stage sea ice			*✓*	with SIN class names

This table constitutes the formalization of the reuse perspective for both reuse scenarios. ppt is parts per thousand. SIN is Sea Ice Nomenclature^15^. Vertical profile means measurements are assigned a depth along the core.

#### Reuse Scenario A

PhD student Ada implemented a 1D physics-based process model of sea ice that simulates heat and salt transport and phase change processes in vertical direction of sea ice such as proposed by Buffo *et al*.^14^. In a next step, she wants to validate the process model by comparing the simulated vertical profiles of sea ice for the state variables temperature, salinity, and solid fraction with measurement data. Sea ice core data is suitable as it often provides several measurements along the core (profile). In order to be useful for the task, measurement data should have the same unit or ratio as the state variables in the model. Ada would like to include uncertainties in the form of measurement errors and standard deviations. She wants to ensure fidelity by only including data with a measurement error below 5%. The extent of the model domain is 1 m, and the spatial discretization is 0.01 m. The distance of measurement locations along a core does typically not coincide with the spatial discretization of the domain. Measurement data may has to be interpolated to match with the location of the computational grid. For a sufficiently good interpolation, Ada restricts the task to measurement data with a mean distance of less than 0.2 m. She needs to know the thickness of the sea ice to ensure sea ice is thinner than the extent of the model domain. Ada would like to use suitable boundary conditions for the model, namely temperature and salinity of the underlying sea water and temperature of the overlaying air. The sea ice upper boundary may be affected from insulating snow covers. Thus, she also needs the thickness of potentially overlaying snow to approximate these effects. The inputs and constraints of Scenario A are listed in Table 1.

#### Reuse Scenario B

Liza is a master’s student. As part of her thesis project she wants to train a classification algorithm based on sea ice core data. She defines the target variables *form* and *development stage* based on the Sea Ice Nomenclature (SIN) provided by the World Meteorological Organization (WMO)^15^. She refers to *form* as the definitions provided in SIN Section 1.1. Sea ice is *fast ice* (1.1.1) if it is attached to the coast. Non-attached occurrence of ice are either *drift ice* or *pack ice* (both 1.1.2). *Drift ice* and *pack ice* are distinguished based on the sea ice concentration. Sea ice concentration above 70% indicates pack ice and below drift ice. She refers to *development stage* as the definitions provided in SIN Section 2 (Development). Section 2 has up to three levels of sub-categories of which Liza considers the first two, referred to as level 1 and level 2, respectively. All sea ice development stages of the first two levels are listed in Table 2. On the level 1, development stage has classes *New ice* (2.1), *Nilas* (2.2), *Pancake ice* (2.3), *Young ice* (2.4), *First-year ice* (2.5), and *Old ice* (2.6). On the level 2 classes, for instance, for *First-year ice* (2.5) are *Thin first-year ice* (2.5.1), *Medium first-year ice* (2.5.2), and *Thick first-year ice* (2.5.3). Sea ice development stage is a property typically assigned by the person(s) who drilled the sea ice core. In the SIN, each of the classes except from *New ice* are assigned with characteristic thicknesses such as ‘30 cm - 2 m’ for *First year-ice* and ‘up to 2.5 m and sometimes more’ for *Second-year ice*. The thicknesses assigned to each level 1 and level 2 of the sea ice development stages are also provided in Table 2. Liza requires the target variables to only consist of classes defined in the SIN. The predictor variables are the mean values of the measurements for sea ice temperature and salinity that have been acquired along the central axis of a core. Furthermore, she wants to include the thickness of sea ice at each coring location, the date of core retrieval as well as the coordinates and the name of the water body, i.e., the sea or ocean, at the coring location. The names of the water bodies should be consistent with the terminology provided by the controlled vocabulary of *The SeaVoX Salt and Fresh Water Body Gazetteer* from the British Oceanographic Data Centre (BODC)^16^. For a quick integration into the training script, she wants the date to be in *YYYY-MM-DD* format and the coordinates to be in decimal degrees. The inputs and constraints of Scenario B are listed in Table 1.

**Table 2:** All sub-categories (2.x refers to level 1 and 2.x.y refers to level 2) of the Sea Ice Nomenclature (SIN) Section 2 Development, which we refer to as sea ice development stages, together with the characteristic sea ice thicknesses as assigned to the definitions in the SIN^15^.

2	Development stage	Sea ice thickness from SIN^15^	Intervals
2.1	New ice	—	—
2.1.1	Frazil ice	—	—
2.1.2	Grease ice	—	—
2.1.3	Slush	—	—
2.1.4	Shuga	—	—
2.2	Nilas	‘up to 10 cm’	[0.0, 0.1]
2.2.1	Dark nilas	‘under 5 cm’	[0.0, 0.05]
2.2.2	Light nilas	‘more than 5 cm’	[0.05, 0.1]
2.2.3	Ice rind	‘to about 5 cm’	-
2.3	Pancake ice	‘up to about 10 cm’	[0.0, 0.1]
2.4	Young ice	‘10-30 cm’	[0.1, 0.3]
2.4.1	Grey ice	‘10-15 cm’	[0.1, 0.15]
2.4.2	Grey-white ice	‘15-30 cm’	[0.15, 0.3]
2.5	First-year ice	‘30 cm-2 m’	[0.3, 2.0]
2.5.1	Thin first-year ice	‘30-70 cm’	[0.3, 0.7]
2.5.2	Medium first-year ice	‘70-120 cm’	[0.7, 1.2]
2.5.3	Thick first-year ice	‘over 120 cm’	[1.2, 2.0]
2.6	Old ice	‘up to 3 m or more’	[0.3, 0]
2.6.1	Residual ice	‘30 to 180 cm’	[0.3, 1.8]
2.6.2	Second-year ice	‘up to 2.5 m and sometimes more’	[2.0, 2.5]
2.6.3	Multi-year ice	‘up to 3 m or more’	[2.5, 4.0]

The sea ice thickness intervals indicates the representation of the development stages in the automatic enrichment routine, which is explained in Step 5.

### Step 1 (b): Reuse scope

Reuse scope is defined by an agnostic data reuser based on the inputs and constraints of the reuse scenario(s). The reuse scope formalizes the desired content of the database. For RESICE, it is the data and metadata required per sea ice core to conduct the scenarios. It should be noted that parts of the reuse scope can be used as a constraint and as an input in the same scenario. For instance, measurement error is an input when combined directly with sea ice temperature for a high-fidelity model validation. At the same time, measurement error is a constraint when only temperature data below a certain error threshold is considered for the validation. Therefore, we will refer to both constraints and inputs uniformly as reuse scope elements. In the following, we will refer to reuse scope also as scope. In this study, scope is combined from Reuse Scenarios A and B as formalized in Table 1. Scope is extended by further elements ID, campaign and polar region to allow filtering of the database. Reuse scope consists of the following elements:

- unique ID of the sea ice core,
- name of associated campaign,
- date, coordinates, name of the polar region and water body of the coring location,
- salinity, solid fraction, and temperature of sea ice assigned with a depth indicating the measurement position along the core (profile) data,
- temperature of the air,
- salinity and temperature of the sea water,
- thickness of snow cover on the sea ice surface,
- thickness of sea ice,
- measurement errors and standard deviations of repeated (profile) measurements,
- mean distances and mean values of the measurements
- form and development stage of the sea ice,
- units of all measurement data and
- naming standards, such as controlled vocabularies, used to classify the water body and form and development stage of sea ice.

The scope is the search target for the content of RESICE. Besides enabling Reuse Scenario A and Reuse Scenario B, a database with this particular scope would furthermore enable other researchers secondary usages.

The granularity of reuse scopes typically implies a structure that does not comply with the content of existing data sets in a one to one relationship. Therefore, the final content and structure of the database may be different from the initially defined reuse scope. Scope elements are assembled from sources in a search process described in the next subsection.

### Step 2 (a): Searching for sources

Source search is initiated on data repositories, where we search for data from sea ice cores reflected in the reuse scope. At best, the reuse scope elements per sea ice core would be provided by one data set alone, and data repositories would have filtering options to allow selection of only those elements that meet defined constraints as defined in Table 1. In reality, data repositories do not provide filtering options for all of the defined constraints, and data sets are not a one by one representation of the reuse scope. Thus, the reuse scope cannot be comprehensively populated from a single data set. The search for missing elements is continued in other data sets and also includes further sources. Sources such as articles and reports provide context for specific sea ice core measurements, and instrument application notes provide general information. We group the different source types and refer to data sets as primary sources, core specific articles and reports as secondary sources and general information as tertiary sources. Table 3 lists all original sources found during the search for sources.

**Table 3:** List of the original sources found during data search.

Primary sources	Secondary sources	Tertiary sources	#Cores
**Pangaea**			
Arndt *et al*.^35^	Arndt *et al*.^36^	WTW (2008a)	21
Katlein *et al*.^59^	Katlein *et al*.^4^*		1
Kramer *et al*.^26^Kramer *et al*.^27^	Kramer *et al*.^57^*Lemke^50^		22
Kramer *et al*.^28^Kramer *et al*.^29^Kramer *et al*.^47^	Kramer *et al*.^57^*		12
Lange *et al*.^30^	Lange *et al*.^45^*	WTW (2008b)	18
Lannuzel^39^	Lannuzel *et al*.^60^Lannuzel *et al*.^52^*	Testo (2024)TPS (2024a)	6
Lannuzel^43^	Lannuzel *et al*.^52^van der Merwe^61^	Testo (2024)TPS (2024b)	5
Lannuzel^48^	Lannuzel *et al*.^52^*Lannuzel *et al*.^49^		7
Lannuzel^42^	Lannuzel *et al*.^52^van der Merwe *et al*.^68^,van der Merwe *et al*.^44^	Testo (2024)TPS (2024b)	9
Lannuzel^51^	Lannuzel *et al*.^52^* Lannuzel *et al*.^69^	TPS (2024b)Testo (2024)	6
Mundy *et al*.^41^	Brown *et al*.^70^*		23
Nicolaus *et al*.^53^Nicolaus *et al*.^54^	Schauer^71^	WTW (2004)	11
Peeken *et al*.^37^	Peeken *et al*.^72^*		5
Pućko *et al*.^56^Pućko *et al*.^63^Isleifson *et al*.^46^	Pućko *et al*.^73^*Pućko *et al*.^74^Isleifson *et al*.^75^	Hach (2000)Control Company (2016)	16
Torstensson *et al*.^33^	Torstensson *et al*.^32^*		14
**Zenodo**			
Audh *et al*.^23^	Johnson *et al*.^2^		21
Omatuku Ngongo *et al*.^24^	Skatulla *et al*.^34^*		15
Wang *et al*.^22^	Wang *et al*.^1^		41
**AADC**			
Duprat^38^	Duprat *et al*.^3^		6
Lannuzel *et al*.^55^			redundant
Meiners^40^	Boebel^62^		28
Trull^58^			redundant

Sources are ordered horizontally by source group and vertically by repository from which the primary source is available. Each row represents the combination of sources that provide fields for one or more sea ice cores. The number of cores that each combination of sources provides to RESICE is listed in the *#Cores* column. Redundant data sets are neglected in RESICE. Secondary sources assigned an asterisk are referenced in the respective primary source. The urls of the instrument manuals listed in the tertiary sources are: WTW (2008a): (https://www.labworld.at/wp-content/uploads/2014/10/Cond_3110.pdf), WTW (2008b): (https://www.labworld.at/wp-content/uploads/2017/09/Bedienungsanleitung-WTW-Cond-3300i-3400i.pdf), Testo (2024): (https://static.testo.com/image/upload/Instruction-manual-and-Software/Instruction-manuals/testo-720-instruction-manual-7808.pdf), TPS (2024a): (https://cdn.shopify.com/s/files/1/0552/9924/4191/files/WP-84.pdf), TPS (2024b): (https://cdn.shopify.com/s/files/1/0552/9924/4191/files/Aqua_C_Manual.pdf),WTW (2004): (https://www.labworld.at/wp-content/uploads/2014/10/Cond_315i.pdf), Hach (2000): (https://www.fondriest.com/pdf/hach_sension5_manual.pdf), Control Company (2016): (https://www.novatech-usa.com/pdf/ControlCompany4000InstructionManual.pdf).

The collection of reuse scope elements per sea ice core is not completed after source search. Instead it is followed by linking relevant parts of the source content with the corresponding scope elements, since not all data and metadata provided in a source are required in the scope. We refer to this combinatorial process as element availability matching. While availability matching is element specific and explained in the next subsection, source search can be generalized and is explained in this subsection.

#### Primary sources

There is a variety of repositories for sharing all types of research results; a selection of general and discipline-specific repositories is provided by Nature Scientific Data (https://www.nature.com/sdata/policies/repositories). We constrained the search for data sets to the three repositories Zenodo, Pangaea, and Australian Antarctica Data Centre (AADC) that majorly publish under licenses that allow reuse and republication, such as CC0, CC BY, or CC BY-SA (https://creativecommons.org/about/cclicenses/). General properties of the data repositories are summarized by the Registry of Research Data Repositories re3data^17–20^ and are reflected in Table 4. Each of the repositories has a different focus and allows for different content types. Pangaea focuses on uploads of geo-referenced tabular data as tab- or xlsx-files but also allows other upload formats (https://wiki.pangaea.de/wiki/Format). AADC allows upload of data sets in common, non-proprietary formats (https://data.aad.gov.au/about/help-and-resources/metadata). Uploads to Zenodo cover all domains, types (e.g., articles, pre-prints, software, presentations) and formats (https://about.zenodo.org/policies/), and uploaded files are directly published without quality control. Submissions to Pangaea and AADC undergo manual checks by data stewards, which can lead to acceptance, revision or rejection. Data repositories request different metadata to be provided by the data collector in the submission mask and alongside or within the data file as summarized in Table 5. The availability of searchable metadata together with query and filter options strongly affects the search on data repositories. None of the repositories provides filter options to select data sets based on constraints discussed in Step 1 (b). Therefore, search is limited to elements, and it often has to be conducted per element as data sets do not provide all elements at once. Some data sets may provide elements of several cores and other data sets provide some elements of one core. Furthermore, not all elements are available as searchable metadata through the search bar.

**Table 4:** General properties of the three data repositories that have been considered for the data search.

	Zenodo^19^	Pangaea^20^	AADC^18^
Focus of data	Research results of all sorts and from all domains	Georeferenced data from the Earth, environmental, and biodiversity sciences	Science data from Australia’s Antarctic research
Repository type	Neither institutional nor disciplinary	Disciplinary	Disciplinary
Content type	Standard office documents Images Plain text Audiovisual data Archived data Source code Scientific and statistical data formats Raw data Other	Standard office documents Images Plain text Audiovisual data Archived data Source code	Standard office documents Images Plain text Scientific and statistical data formats
Quality control	None	Manual by data steward	Manual by data steward

The content is provided from the Registry of Research Data Repositories^17^.

**Table 5:** Submission and search properties of the data repositories.

	Zenodo	Pangaea	AADC
Fields in data submission mask	Title* Description* Resource type*(Publication, image, code, etc.) Publication date* Creators* License Contributors Keywords Languages Dates Version Publisher etc.	Title* Description Authors* Keywords License* References Projects Grants	Title* Description* Submission type* (New data or replacement) Release status* (Public, embargoed, review, etc.) Project Meta data record*
Further metadata to be provided with data set	—	Date or time* Coordinates* Corresponding publication Project Parameter units Instruments Methods etc.	Temporal coverage Spatial coverage Purpose Quality Access Science keywords Additional keywords Locations Platforms Instruments Researchers Use constraints etc.
Use of vocabularies or ontologies	—	Parameter names linked to, e.g., Environment Ontology, dbPedia, Wikipedia, reference height	Global Change Master Directory (GCMD) keywords
Data set search filter options	Access status Resource types Subjects File type	Date and coordinate coverage Author Basis (Vessel, land) Publication year Topic Projects Method or device Campaign Location (in words)	Date coverage Coordinate coverage Researchers Source (Vessel, laboratory, field) Keywords
Searchable metadata	Advanced search based on field name metadata	Advanced search based on field names and further metadata	Simple search through metadata content

Fields annotated with an asterisk were clearly obligatory. For Zenodo, options for the advanced search can be found in the search guide (https://help.zenodo.org/guides/search/). For Pangaea, the metadata required for submission (https://wiki.pangaea.de/wiki/Data_submission), the reference height (https://wiki.pangaea.de/wiki/Geocode), and the advanced search options (https://wiki.pangaea.de/wiki/Pangaea_search) can be found in the Pangaea wiki. For AADC, we added further metadata not mentioned in the submission mask based on metadata from existing data sets. It was not clear to us which further metadata is obligatory for AADC in the DIF format metadata record.

In Zenodo, our search for *sea ice salinity* with filter options *access* set to *open* and *resource type* to *data set* has around 9500 results. The first search result was a data set from Oggier^21^, which provides data from lab experiments; it is excluded. The subsequent three results^22–24^ contain elements of the scope and are therefore selected. More data sets from Zenodo are not included as the subsequent search results are either laboratory data, 2D satellite data, or modeled data. Other combinations of elements in the search query did not improve the first results. Searchable metadata is generated through Zenodo’s submission mask and is not directly linked to actual data file content. Each data file has to be manually checked for availability of the elements advertised in the metadata.

In Pangaea, advanced search for *sea ice, parameter:salinity AND parameter:temperature AND parameter:depth ice/snow* and more granular element-wise versions yields around 230 results. All predefined keywords (e.g., parameter, method, author) that can be used in the search are consistent with the fields in the data file due to *relationalization*^25^, so that *parameter:salinity* provides data sets with a *salinity* column. A manual check to verify that the data described in the metadata is available in the data file is not necessary, and the query options allow a specific search for a combination of elements. The provided filter options for the search results were not useful to further restrict the results. It should be noted that Pangaea data sets with names Kramer *et al*.^26–29^ each represent several sources. They are all indicated in the references. The same holds for Lange *et al*.^30^.

In AADC, search for *sea ice salinity temperature* yields around 1000 results. Special about AADC is that a metadata record is created separately for each submitted record using a metadata creation tool, which is assigned to the respective data set during the submission process. The metadata record has Directory Interchange Format (DIF) format and uses Global Change Master Directory (GCMD)^31^ keywords. The content of the metadata record is findable via the search bar. Yet, the keywords defined in the DIF file may differ from the actual label names in the data files, so they have to be manually cross-checked for consistency with the metadata.

#### Secondary sources

Data sets are often published as supplements to articles. Such articles provide context to the measurement data and may contain missing elements of the reuse scope. In Pangaea, articles are often directly linked in the data set, such as the article Torstensson *et al*.^32^ in the corresponding data set Torstensson *et al*.^33^. The Zenodo data set from Omatuku Ngongo *et al*.^24^ provides a reference to an article by Skatulla *et al*.^34^ in the description of the accompanying PDF file. If no such resource is referenced in the data set, a google search of the campaign name and the data set authors may reveal related articles. For example, the data set Arndt *et al*.^35^ does not reference a specific article, but a google search for the campaign name *PS 118* and the first author’s name *Arndt* reveals a journal article by Arndt *et al*.^36^ that describes *PS118*. In addition to articles, there are expedition reports, which provide an overview on entire measurement campaigns and may contain reuse scope elements missing in the data sets. The electronic Publication Information Center (ePIC) is an official repository of the Alfred Wegener Institute (AWI) and publishes its expedition reports.

#### Tertiary sources

Elements still missing after searching in primary and secondary sources may be matched from sources unrelated to the specific sea ice core measurements. Tertiary sources are mostly instrument manuals that provide specifications of the instruments. Manuals can be found by a google search of the instrument names or by searching in the manufacturer online shops. Other tertiary sources used in this article are naming standards. They are the Sea Ice Nomenclature, which provides definitions for sea ice and is findable in the World Meteorological Organization’s e-Library, and the *The SeaVoX Salt and Fresh Water Body Gazetteer* (SeaVoX) from the British Oceanographic Data Centre (BODC)^16^, which is a controlled vocabulary for water body names. The latter provides the shapefile *Polygon data set of the extent of water bodies* delineating Earth’s water bodies into distinct polygons, each tagged with attributes defining its respective ocean or sea. As tertiary sources are independent of specific sea ice cores, matching elements from tertiary sources uses elements already matched from primary or secondary sources. For example, to find a missing measurement error of an instrument the name of this instrument has to be available from primary or secondary sources in order to find the corresponding manual (tertiary source).

### Step 2 (b): Matching available elements

**Fig. 2:**
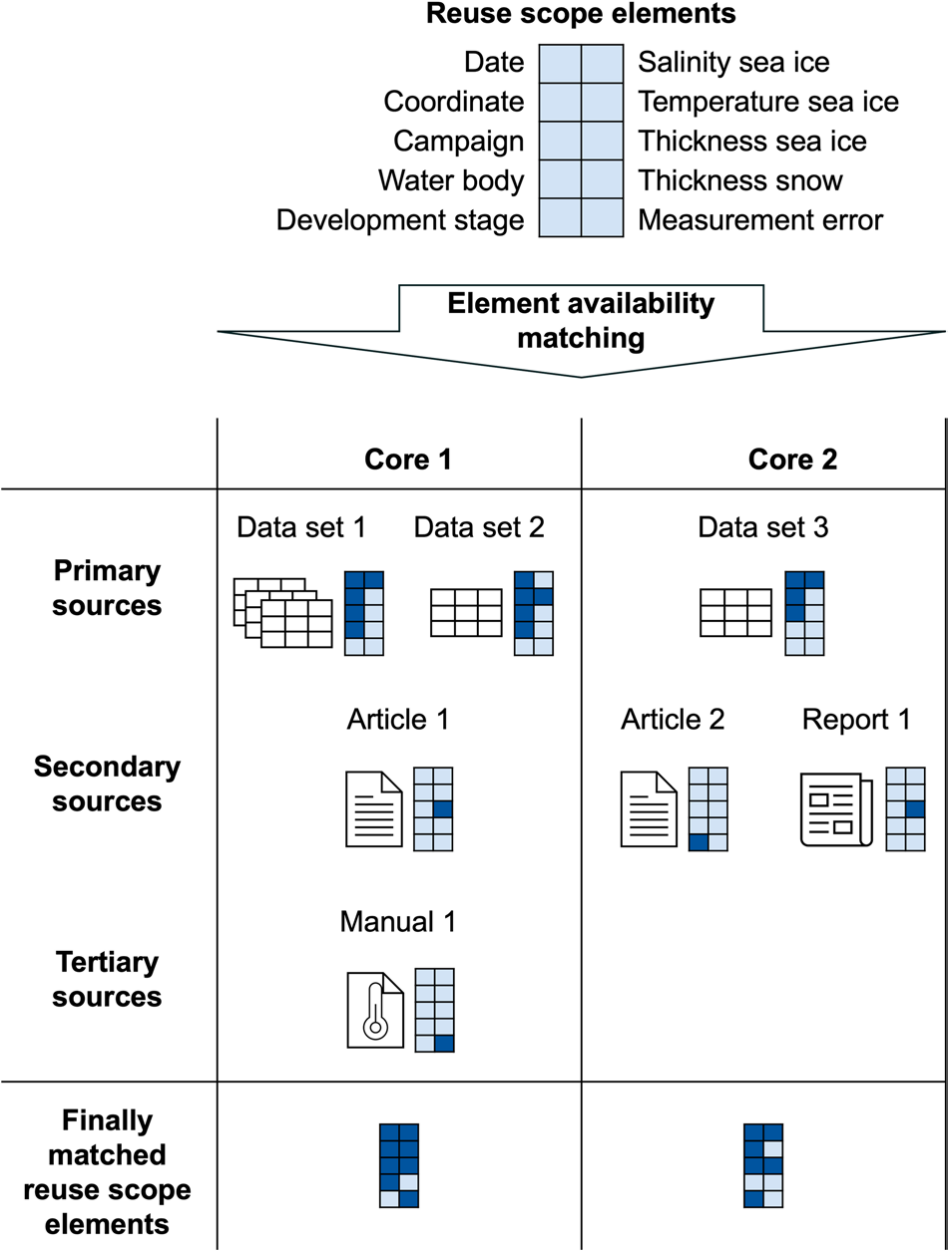
Element availability matching for two exemplary sea ice cores based on a reduced reuse scope w.r.t. Table 1. The ten light blue boxes at the top represent the reuse scope elements. During element availability matching, reuse scope elements are matched by searching first in primary and then in secondary and tertiary sources. Dark blue boxes represent matched elements.

Element availability matching is necessary due to significant mismatches between structure and content as anticipated in the reuse scope and as actually available from sources. It cannot be assumed that the elements of interest are comprehensively available in data sets. Instead reuse scope elements have to be combined from different sources for each sea ice core, as illustrated for a reduced reuse scope in Fig. 2, before integrating them in RESICE. Through element availability matching data and metadata from sources are first identified as relevant for the reuse scope and then linked to the respective reuse scope elements for each sea ice core. Any element that can be matched from a source is a hit. We distinguish between direct and indirect hits. Direct hits occur when the relation of a source to a reuse scope element is unambiguously understandable to the reuser, such as a column in a data file, in the data set’s metadata, or in a table from a secondary source. The respective data or metadata has to be assigned with explicit, easy to understand, labels. Hits are indirect when additional common sense or context has to be applied for the matching, such as missing or not explicit labels, or in case a graphic provides the respective data or metadata. Availability depends on the source group from which the element is matched. Primary sources are direct sources and secondary and tertiary sources are indirect sources. Direct hits with direct availability are desired.

**Fig. 3:**
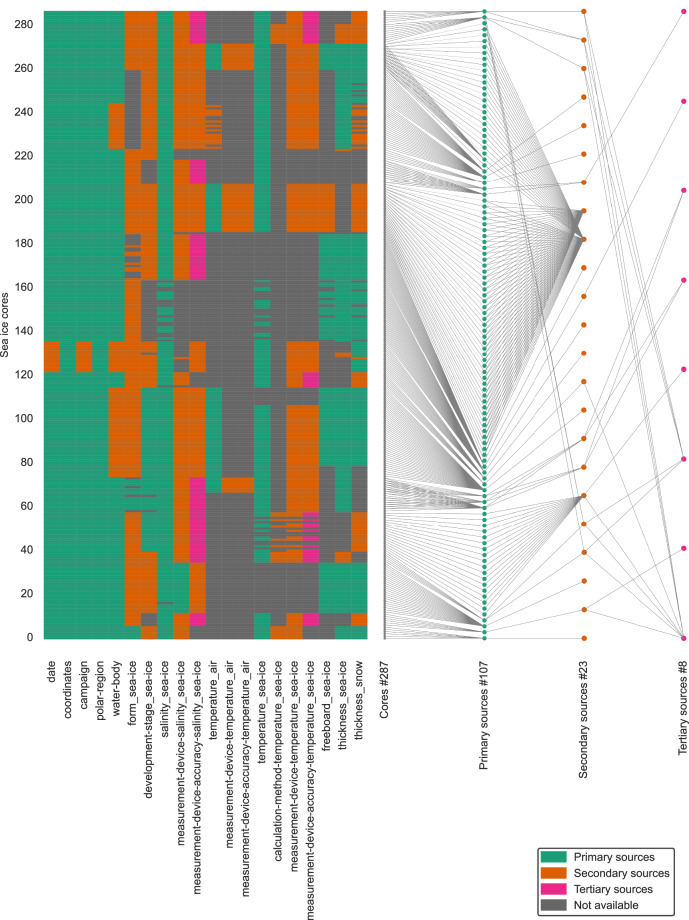
(**a**) Overview on the availability of the fields in each YAML file per sea ice core (y-axis) of the RESICE extendable database. Green, orange and pink refer to availability from a primary, secondary or tertiary source, respectively. Gray means the field is not available. (**b**) Data2source traceability plot for RESICE. Each gray node represents one YAML file or sea ice core. An interactive version of this figure is available (10.5281/zenodo.14916241).

Availability matching is a sequential and iterative process. It is sequential because the matching process begins by finding a data set that provides elements for a core or selection of cores. This data set together with further sources is used to find as many elements as possible for this core or selection of cores. Only then does the matching process begin for a new core or selection of cores from another data set. The process is iterative because more scope elements are matched by moving from primary to secondary to tertiary sources. It should be noted that the matching process is subjective as it depends on the reuser’s search method and the sources considered. An element may appear to be missing from the sources, but it may eventually be available from a source that remained undiscovered by the reuser. Scope elements are available as defined, have to be adjusted to fit the scope or are unavailable. Further elements may be added during availability matching; they have not been anticipated in the reuse scope but are available from the sources and add value to the database. The checkerboard in Fig. 3(a) shows the availability per element and per sea ice core. In the following, we discuss availability matching separately per reuse scope element as listed in Step 1 (b). The matching process is carried out manually. An automation of the process is challenged by the heterogeneity of the sources, which requires interpretation within the context and core specific sources of interest. The manual matching process is complemented by an automatic enrichment process for elements that are not available as required from the sources. This process is described in detail in Step 5.

#### ID

The ID is a sea ice core-specific name or number used to differentiate sea ice cores. Usually data sets provide a name or a number for each core and location, also called station, in the metadata or as a specified column entry. We compose IDs based on the provided information.

#### Campaign

The name of the campaign, expedition, cruise, or project is available from primary sources for ~95% of the cores. Direct hits occur when Pangaea’s metadata label *Campaign* or other repository data sets have labels such as *Cruise* as in Zenodo data sets Audh *et al*.^23^ and Omatuku Ngongo *et al*.^24^. In all other situations with direct availability, the campaign name is also a direct hit but from the data set’s general information in form of unlabeled metadata (e.g., Wang *et al*.^22^). For the remaining ~5% of the cores, which are all sea ice cores from Torstensson *et al*.^33^ data set, campaign is indirectly available from contextual information provided in the secondary source by Torstensson *et al*.^32^.

#### Date

Date is available from primary sources for ~95% of the cores. It is often a direct hit as a column in a data file (e.g., Omatuku Ngongo *et al*.^24^), metadata label (e.g., Kramer *et al*.^28^) or both (e.g., Peeken *et al*.^37^). Pangaea data sets always provide date as metadata except from the data set Torstensson *et al*.^33^, where date is indirectly available as direct hit from a table with column label *Date* provided in the accompanying secondary source^32^. The AADC data set from Duprat^38^, provides the column label *Julian Day* in the data file, which would result in an indirect hit because it has to be transformed into a date first. The data set’s metadata, however, provides the date so that it is matched as direct hit from there. Secondary sources usually provide the date, when describing the sea ice cores.

#### Coordinates

Coordinates are always provided with direct availability. Most of the hits are direct as data file column labels *Longitude* and *Latitude* in Omatuku Ngongo *et al*.^24^ or *Location* in Wang *et al*.^22^. For all Pangaea data sets, coordinates are provided in the metadata with labels *Longitude* and *Latitude* (e.g., Lange *et al*.^30^). Coordinates can also be redundant, when available in the metadata and in the data file, which is the case for Lannuzel^39^. Meiners^40^ data set consists of several data files in xlsx-format each containing data from different measurements. Instead of directly providing coordinates for the measurements, the names and numbers of the station are assigned per measurement. One data file *PS117_IceStations_Positions_PS117* provides station names *Station* and station numbers *St#* assigned with coordinates *Lat S* and *Long W*. The data file *PS117_IceStations_Chlorophyll_a* providing salinity measurements contains columns named *Station* and *Site*, and the data file *PS117_Ice_Temp_Profiles* providing temperature measurements assigns a *Core_Site* for each core, and it also provides unlabeled comments for each core, e.g., *Ice1*, *Ice2*. Salinity measurements can be linked to coordinates with the *Station* column in form of a direct hit. The linkage of temperature measurements with the corresponding coordinates is ambiguous since the entries for *Core_Site* do not coincide with the entries for *Station* or *St#*; coordinates cannot be easily linked. After closer assessment, we assume that the mentioned unlabeled comments per core such as *Ice1* refer to the station numbers provided in column *Station*. We match the coordinates accordingly as indirect hits. Temperature and salinity data of the data set Meiners^40^ is included as separate cores in the database, since we cannot verify whether the cores were taken in close proximity or not.

#### Polar region

Polar region is always directly available. Most cores have Arctic or Antarctic in the data set title (e.g., Torstensson *et al*.^32^; Wang *et al*.^22^) or the data set description (e.g., Omatuku Ngongo *et al*.^24^; Duprat^38^, so it is a direct hit. In all other cases, the polar region can be derived from the coordinates, so it is an indirect hit with direct availability.

#### Water body

The name of the sea or ocean, from where the core was retrieved, is provided with direct availability for ~73% of the sea ice cores. Direct hits occur with metadata label *Location* for data sets from Pangaea and AADC, which is assigned a name for the water body such as *Southern Ocean*^40^, *Scotia Sea*^26^ or *Lincoln Sea*^30^. For the other cases, the name of the water bodies are indirectly available as indirect hits from accompanying papers such as for the sea ice cores from the data sets Torstensson *et al*.^33^ and Wang *et al*.^22^, which are matched from maps provided in the articles Torstensson *et al*.^32^ and Wang *et al*.^1^, respectively. The water body of the sea ice cores from the data set Duprat^38^ is missing. Information on the use of standardized naming schemes for the water bodies, for instance, in form of controlled vocabularies is not provided for the agnostic reuser.

#### Salinity sea ice

Salinity of the sea ice, along with the depths of bottom and top or center of the respective section (salinity is measured from melted section of the core), is directly available for ~95% of the cores. For the remaining ~5% of the cores, salinity measurements were not available. The hits are mostly direct because of the distinctive label names for salinity sea ice such as *Salinity/PSU*^22^, *Ice bulk-salinity*^40^, *Salinity (psu)*^38^, *Salinity*^39^, and *Sea ice salinity*^35^. The depth label for temperature measurements is often *depth ice/snow*, and it can also be *Av. Depth (cm)*^38^, or *Ice depth [m]*^24^. The depth label corresponding to salinity sea ice is ambiguous in data sets Meiners^40^ and Torstensson *et al*.^33^, where it is labeled *HPLC Core* and *ice thickness*, respectively. These are indirect hits since additional context was used for the matching.

#### Solid fraction sea ice

The solid fraction of the sea ice is not a typical measurable. It is therefore not available and missing for all sea ice cores. The reader should note that solid fraction is available in some data sets, where it is post-processed as a function of salinity sea ice and temperature sea ice. Since it is not a measured value, we neglect it.

#### Temperature sea ice

Temperature of the sea ice, along with its measurement depth, is available from primary sources for ~73% of the cores. Most of the hits are direct because of the distinctive label names for temperature such as *Temperature*/°*C*^22^, *t* [°*C*]^24^ and *Temperature, ice/snow*^39^. Depth labels are similar to those listed for salinity sea ice. A special case is the data set from Torstensson *et al*.^33^, where the label name for temperature is *Temperature, water*. However, we understand them as temperature sea ice since the values range between -0.25 and -2.65°C with the lower temperature being too cold for seawater to stay liquid. Furthermore, the corresponding article^32^ describes the measurement of sea ice temperature directly after core retrieval, but it does not mention temperature measurements of water. Furthermore, the article describes that brine salinity is computed using temperature sea ice and salinity sea ice, which also implies it is the temperature of the ice. Thus, we match temperature sea ice as an indirect hit from the column *Temperature, water*. For the remaining ~27% of sea ice cores, temperature is not available. This is the case for Arndt *et al*.^35^, Mundy *et al*.^41^ and some of the cores from Meiners^40^.

#### Calculation method temperature sea ice

In some cases the temperature data provided for the sea ice cores is linearly interpolated in the data sets. This detail was revealed during the search process and is therefore added to the reuse scope. We found two reasons for linear interpolations. First, temperature measurement data in the sea ice may be linearly interpolated to match the locations of the salinity measurements. This is the case for data sets from Lannuzel^42,43^ and Duprat^38^, and it is described in the respective articles^3,44^. Second, temperatures may be linearly interpolated between one measurement at the surface and an estimate of the temperature at the sea ice bottom. This is the case for the data set from Lange *et al*.^30^, and the procedure is described in the accompanying article Lange *et al*.^45^. It is important to note that information on linear interpolation was never directly available from a data set. Instead it is only indirectly available through indirect hits in secondary sources.

#### Temperature air

Temperature of the air is not available for ~66% of the cores. For ~29%, it is directly available. The data set from Wang *et al*.^22^ provides air temperature for each sea ice core as a direct hit with direct availability with label *Air temperature* (°*C*). Isleifson *et al*.^46^ and Kramer *et al*.^27^ provide air temperature with label *temperature, air*. For ~5% of the sea ice cores, which are all from data set Audh *et al*.^23^, air temperature is provided with indirect availability from the secondary source Johnson *et al*.^2^, where it is provided in a table as direct hit. We include air temperature whenever it was available.

#### Temperature and salinity of the seawater

Both elements are unavailable for the sea ice cores considered.

#### Thickness snow

Thickness of snow cover on top of the sea ice sea is directly available for ~51% of the cores. It is a direct hit, for instance, with labels *snow thickness*^35,41,47^ and *Snow [m]*^40^. In data sets Duprat^38^ and Torstensson *et al*.^33^, snow thickness is an indirect hit because it has labels *Av. Depth [m]* and *Section* and *Depth ice/snow*, respectively, so that context had to be used for matching. Thickness of snow is indirectly available for ~28% of the cores. It is a direct hit for sea ice cores from Lannuzel^48^, where it is matched from a column with label *Snow* provided in the paper Lannuzel *et al*.^49^ and for sea ice cores from Audh *et al*.^23^, where it is directly matched from a table with column label *Snow depth (cm)* provided in the paper from Johnson *et al*.^2^. For the sea ice cores from Kramer *et al*.^26,27^, snow thickness is an indirect hit with indirect availability as it is measured from a graphical representation of the sea ice cores provided by the expedition report by Lemke^50^. It is not clear to agnostic reusers whether the absence of snow thickness data is equivalent to a missing snow cover.

#### Thickness sea ice

Thickness of sea ice is directly available for ~64% of the cores. It is a direct hit with direct availability for data sets Mundy *et al*., Kramer *et al*., and Wang *et al*.^22,41,47^. Sea ice thickness in data set Arndt *et al*.^35^ is the sum of snow thickness and sea ice thickness, so that first snow thickness has to be subtracted; it is an indirect hit with direct availability. Sea ice thickness is labeled *total core length* in the data set from Meiners^40^ as explained in the provided readme file, which is an indirect hit. Sea ice thickness is indirectly available for ~5% of the cores. Direct hits from secondary sources are for sea ice cores from Lannuzel^48^ since they are available from a table in the paper Lannuzel *et al*.^49^. The article Torstensson *et al*.^32^ provides sea ice thickness as a direct hit with indirect availability for cores from the data set Torstensson *et al*.^33^. Sea ice thickness is missing in data set Lannuzel^51^, but it can be measured from a graphical representation of sea ice in the article from Lannuzel *et al*.^52^ not referenced in the data set. For the sea ice cores from data sets Kramer *et al*.^26,27^, sea ice thickness is an indirect hit with indirect availability. It is measured from a graphical representation of the sea ice cores provided in a chapter of the corresponding expedition report by Lemke^50^. Sea ice thickness is missing for several sea ice cores such as from Nicolaus *et al*.^53,54^ and Audh *et al*.^23^. Missing values of sea ice thickness were (manually) filled with the deepest measurement location (~31%) from the salinity or temperature profile, which is an indirect hit with direct availability. For the sea ice cores from Duprat (2019), sea ice thickness is not explicitly reported. Figure 2 in the corresponding article Duprat *et al*.^3^ shows the measurement locations along the core. The lowest measurement is 2.5 cm above the ice water interface. Therefore, 2.5 cm was added to the lowest measurement of each core.

#### Freeboard sea ice

Sea ice thickness is often accompanied with sea ice freeboard, which is the extent of sea ice above the water level. Sea ice freeboard is added to the reuse scope since it could be useful for other reuse scenarios. It is available as direct hit with direct availability (~40% of the cores) in data sets Mundy *et al*.^41^, Kramer *et al*.^47^, Meiners^40^, Arndt *et al*.^35^ and Wang *et al*.^22^. For the sea ice cores from data sets Kramer *et al*.^26,27^, sea ice freeboard is a direct hit with indirect availability as it is available in a table of the secondary source by Lemke^50^.

#### Measurement error

Measurement errors for salinity and temperature measurements are not available. Primary sources from AADC discuss reasons for uncertainties in the measurements such as Lannuzel *et al*.^55^ or outliers such as Duprat^38^. However, the context and related impact on the measurement error is neither quantifiable nor sufficiently interpretable to be used meaningfully by data reusers.

#### Instrument accuracy

While a general measurement error is not available, instrument accuracy is available, and it is added to the reuse scope. Instrument accuracy is never directly available. For salinity sea ice, it is provided as direct hit from secondary sources for ~49% of the cores, and for temperature sea ice it is ~27%. In these cases, instrument accuracy is often combined with the name of the instrument. For ~28% of the cores, instrument accuracy of salinity, and for ~16% of the cores, instrument accuracy of temperature sea ice is matched from the respective instruments manual.

#### Instrument name

Measurement error is not available as such and instead it is replaced by instrument accuracy. In some cases, a secondary source would provide the name of the instrument but not the accuracy. Therefore, we add the name of the instrument to the scope, so that it is documented for a subsequent search for accuracy specifications in tertiary sources. The names of the instruments used to measure salinity and temperatures are never available from primary sources except from the sea ice cores provided in Mundy *et al*.^41^ data set, which contains the name of the salinity measurement device. For the majority of sea ice cores, the instrument for salinity sea ice (~78%) and temperature sea ice (~86%) measurements is indirectly available as direct hit, and for the rest it is not available.

#### Standard deviation

Standard deviation for repeated measurements of salinity and temperature measurements is never available. Wang *et al*.^22^ data set is the only primary source that provides standard deviation, in this case for sea ice thickness; it is included in the database. Meiners^40^ data set provides repeated measurements of snow thickness without inferring standard deviation. The standard deviation is calculated for Meiners^40^ before it is integrated in RESICE.

#### Mean distances of temperature and salinity sea ice

The mean distances of measurement locations along the core of the temperature and salinity of sea ice is never available as metadata.

#### Mean values of temperature and salinity sea ice

The mean values of temperature and salinity of sea ice is never provided except from Wang *et al*.^22^ data set.

#### Form and development stage sea ice

The elements sea ice form and development stage are often available in a combined form or with similar label names. Therefore, they are searched for together during availability matching. Next, they are disentangled where necessary into two independent elements. Development stage is available from primary sources for ~25% and from secondary sources for ~58% of the cores. For sea ice form, it is ~6% and ~75%, respectively. Indirect hits with direct availability are matched from the column name *ice type* for Pućko *et al*.^56^, where it is a combination of both elements *landfast first-year ice*, for Wang *et al*.^22^, where it is equivalent to sea ice development stage, and for Peeken *et al*.^37^, where it is equivalent to sea ice form. Furthermore, Lannuzel^42^ provides both elements in a combined form in the comment section, namely *first year pack ice (granular columnar)*. The data set from Duprat^38^ reports that the data represents *land fast sea ice*. Indirect hits with indirect availability are, for example, sea ice development stage for the cores from Audh *et al*.^23^. In this case, sea ice development stage is matched from information provided in the paper from Johnson *et al*.^2^, where it is called *first-year Antarctic sea ice*. Sea ice form and development stage for the data sets from Kramer *et al*.^28,29^ are also matched from the accompanying paper Kramer at al.^57^, which states sea ice was *first-year ice* and that all cores were *drifting pack ice* except from *IO-5* which was *offshore fast ice*. Furthermore, the use of terms for sea ice development stage often mixes categorical levels of the SIN. Wang *et al*.^22^ use *first-year ice*, which is level 1 (sub-category *2.5)*, and *multi-year ice*, which is level 2 (sub-category *2.6.3)*, to define their *ice type* column.

When sea ice form is missing, the SIN can be used to manually fill the element (~17%) by interpreting sea ice form based on sea ice concentration if available. In this manner, the SIN was used for sea ice cores from the data set Wang *et al*.^22^. The accompanying paper by Wang *et al*.^1^ states that the cores were taken from *vast ice floes* that had *diameters of several kilometers*. Therefore, we assume sea ice concentration to be above ~70%, which is consistent with sea ice form *pack ice*. The same holds for the data set Audh *et al*.^23^ data for which locations the accompanying paper^2^ shows a map of sea ice concentration. In this map, all coring locations appear to be above ~70%.

Lastly, none of the data sets refers explicitly to the Sea Ice Nomenclature (SIN) or other naming standards. Only in the article by Skatulla *et al*.^34^, we found a reference to a standard. They state ‘With reference to WMO (code 3739) ice age ID 5 applied for the southernmost ice station and ice age ID 3 for the most northerly station.’ The mentioned *WMO (code 3739)* defines the development stage of sea ice (https://artefacts.ceda.ac.uk/badc_datadocs/surface/code.html). Accordingly, ID 3 refers to ‘predominantly new and/or young ice with some first-year ice’ and ID 5 to ‘all thin first-year ice (30 - 70 cm thick)’.

#### Units and ratios

Data sets usually provide units together with the measurement data. This can be in form of the *Unit* column in Pangaea data sets or combined with the column label in data sets from Zenodo and AADC such as *Depth (m)*, *Ice Temp°* and *Snow (cm)* in Meiners^40^, *Av. Depth (cm)*, *Temperature. (oC)* and *Salinity (psu)* in Duprat^38^ or *Ice depth [m]* and *sal [PSU]* in Omatuku Ngongo *et al*.^24^. The ratio for salinity is often not mentioned in the data sets. In this case, we assume it to be ratio parts per thousand (ppt).

#### Naming standards

AADC metadata records^38,40,55,58^ use Global Change Master Directory (GCMD) keywords to specify the location and the water body. Pucko *et al*.^56^ provide their classification scheme used to classify *ice type* column of their data set, which is a combination of *development stage sea ice* and *form sea ice*. The paper from Arndt *et al*.^36^ provides the classification scheme for the column *ice age classification*, which is similar to the development stages classes names from SIN. Furthermore, the article by Skatulla *et al*.^34^ refers explicitly to the SIN to classify the sea ice development stage as described above. We did not find references to other naming standards.

### Step 3: Plausibility checks of the sources

We made several observations in the original sources that required special attention for consistent integration into RESICE. As a result, some original sources were omitted or had to be adapted. We identified several challenges that are described in the following sections.

#### Redundant data sets across repositories

The same data sets may be available from different repositories. We found that the Pangaea data sets Lannuzel^42,51^ appear to be duplicates of the AADC data sets Trull *et al*.^58^ and Lannuzel *et al*.^55^ as they provide equivalent measurements for the same coordinates and the same campaign. The data sets do not reference each other. The measurement values are duplicated, but there are differences between the repository entries. For example, the sea ice core with ID *SIPEX-01* has one more temperature sea ice measurement in the Pangaea data set, while the AADC data set provides more context on data quality. Furthermore, the Pangaea data sets Kramer *et al*.^28,29^ appear to be redundant with Pangaea data set Lannuzel^42^ as they all provide data for the same campaign name, namely *SIPEX*, and dates. However, a closer inspection shows that the coordinates and measurement values are not equivalent between the data sets. Consequently, it is assumed that different measurements took place during the same cruise. All three data sets, Kramer *et al*.^28,29^ and Lannuzel^42^, are included in the database.

#### Duplicates within the same data set

Equivalent measurements may appear duplicated in the same data set. One example is the data set from Torstensson *et al*.^33^, which has repeated measurements for *Fucoxanthin concentration* at the same location. Salinity and temperature measurements are not repeated. Instead, they seem to be duplicated in the same data set and per Fucoxanthin measurement. In this case, temperature and salinity data is only included once per location in the database. Another example is Lannuzel *et al*.^48^, where cores *05*, *06*, and *07* are assigned equivalent temperature measurement data, and cores *07* and *08* are assigned equivalent salinity measurements. Here, we keep all data, as it is not clear from which location the measurements originate from. Omatuku Ngongo *et al*.^24^ assign equivalent snow thickness measurements to repeated salinity and temperature measurements from the same location. In this case, we keep all snow thicknesses and add them to the database RESICE. Katlein *et al*.^59^ also provide potential duplicates in the data set. Each *Depth ice/snow* value is duplicated except from the first and the last ones. The measurement values seem doubled but shifted by one depth value. We used each measurement value only once.

#### Incorrect metadata

The metadata provided in a data set may be incorrect. This is the case for the name of the salinity measurement device in Torstensson *et al*.^33^, which is provided as *Cond 310i* from manufacturer *WTW*. While searching for the related instrument accuracy via google and the manufacturers homepage and consulting the costumer service of WTW, we found out that there exist *Cond 315i* and *Cond 3110* but not *Cond 310i*. Skatulla *et al*.^34^ also provide a non-existing instrument for temperature measurements for Omatuku Ngongo *et al*.^24^, which is called *GMH 3750-GE logger* from manufacturer *Testo*. However, this instrument cannot be found via google nor the manufacturers homepage. Instead an instrument with this name is available from the manufacturer *Greisinger*. We changed the name of the manufacturer before integrating the cores into RESICE.

#### Inconsistencies within the same data sets

In the data set by Meiners^40^ data and metadata is stored in separate files, and the connection between them is ambiguous due to inconsistent naming. It is not clear if salinity and temperature measurements originate from the same core, individual cores in close proximity, or individual cores at different locations. Temperature and salinity measurements are therefore separately integrated in the database RESICE to differentiate them. In the data set from Mundy *et al*.^41^, we found an inconsistency in the depths that are assigned to the salinity measurements. Salinity measurements are made for melted sections of a core, so that one salinity measurement is associated with two depths, one at the top and one at the bottom of each section. We use both depths values to compute the center of the section, which is the depth assigned to the salinity measurement in the RESICE database. In Mundy *et al*.^41^, the depth for the top of the section often has a higher value than the bottom of the section, for instance, it is 0.900 m for the top and 0.110 m for the bottom for one of the cores. We assume that the bottom depth value has a typo and should be 1.100 m. The center of the section is calculated with the corrected value before integrating them in the RESICE database. We found another inconsistency in the data sets by Lannuzel^39^,^43^,^51^, where thickness of sea ice and snow as well as sea ice form and development stage are provided as metadata in the comment section. After cross-checking with the measurement data, we found that values for sea ice thickness from the comment section are implausible as the measurement depths go beyond the provided sea ice thickness. Furthermore, the assigned development stages are inconsistent. Consequently, the comment sections of these data sets are neglected.

#### Inconsistencies between sources

For many sea ice cores more than one source provides relevant data and metadata, and there may arise inconsistencies between these sources. This could be, for instance, a naming inconsistency making the connection between the sources difficult. This is the case for the names of the sea ice cores in the data set from Lannuzel^39^ and the accompanying paper Lannuzel *et al*.^60^. We detected this inconsistency through sea ice thickness values from Table 1 in the paper, which did not fit to the measurement depths of the respective cores from the data set. Apparently, there was a mix-up in the naming, so that sea ice cores *V*, *IX*, and *VII* from the paper are equivalent to *IX*, *VII*, and *V* in the data set. We used the naming of the paper. Another naming inconsistency was found between data set Lannuzel^43^ and paper by van der Merwe *et al*.^61^, where we matched sea ice core *XX* from the data set with data and metadata such as sea ice thickness from sea ice core *10* in the paper. Other inconsistencies include different dates in different sources for the same core. For example, Lannuzel^42^ assigns 17/09 to sea ice core *05*, while the paper by Lannuzel *et al*.^52^ assigns 18/09. We use the date from the data set. Another inconsistency concerning sea ice form was found for the cores from Meiners^40^ from campaign PS 117. The corresponding expedition report states that 5 of the 8 ice stations were located in the *Eastern Weddell sea*, which was a *pack-ice zone*, and 3 stations were located in the *Western/North-Western Weddell sea*, which was a *marginal ice zone*^62^. However, after checking all locations on the map 8 of the sea ice core locations seem to lay in the eastern Weddell Sea. Another example is an inconsistency for the sea ice development stages for the sea ice cores from the data set Kramer *et al*.^26,27^. The accompanying paper Kramer *et al*.^57^ defines sea ice development stage for the four sea ice cores *WS-4*, *WS-7*, *WS-11*, and *WS-21* as *multi-year ice* and the rest as *first-year ice*. The corresponding expedition report by Lemke^50^ provides sea ice development stage for each date in a table, which assigns *WS-21 first-year ice* while *WS-4*, *WS-7*, *WS-11* are all *second-year ice*.

#### Misleading semblance of accuracy

The given decimal places of a measurement may exceed the actual possible accuracy imposed by the instrument. For example, in the data set from Lange *et al*.^30^, sea ice salinity and temperature are provided with three decimal places. The instruments used have accuracies of 0.1 for salinity and 0.2° for temperature. The same holds for Torstensson *et al*.^33^, who provide sea ice temperature with 3 decimal places although the reported accuracy of the measurement device is 0.1°. Also the data sets from Lannuzel^39,42,43,48,51^ give two decimal places for sea ice temperature and three decimal places for sea ice salinity, although the accuracies of the salinity instruments used are 0.4 ppt and 0.06 ppt and the accuracy of the temperature instrument is 0.2°. In this case, all measurement values are rounded to 1 decimal place before integrating them into RESICE.

#### Reference depth unclear

The reference depth of the measurements along the central axis of the sea ice cores is not always clearly defined. In RESICE, reference depth of 0 m should be at the interface of snow and sea ice. Measurements in the sea ice should be defined a positive value. Data sets Omatuku Ngongo *et al*.^24^, Audh *et al*.^23^ and Arndt *et al*.^35^ define reference depth as requested and assign measurements in the sea ice a positive depth. In some data sets reference depth is not explicitly defined, but it can be derived from the context. For Duprat^38^, measurements in the snow or the sea ice are consistently assigned a positive or negative depth, respectively. We switched the signs of the depth values assuming reference depth is again at the snow sea ice interface. Wang *et al*.^22^ do not explicitly define reference depth. Since the reported sea ice thickness is equivalent to the respective deepest sea ice core measurement, we conclude that the reference depth is at the requested position. Meiners^40^ does not define reference depth but always provides snow thickness measurements. Thus, we concluded reference depth is again at the interface of sea ice and snow. Lannuzel^48^ does not define reference depth, but the accompanying paper by Lannuzel *et al*.^49^ provides a table, where snow thickness is assigned a negative depth. Therefore, we assume reference depth is as requested. The majority of data sets do not define the reference depth. In these data sets^26–30,33,39,41– 43,51,54,59,63^, we assume reference depth of 0 m to be at the intersection of snow and sea ice. The reader should note that Pangaea data sets such as Peeken at al.^37^ and Katlein *et al*.^59^ link the measurement depth along the core *depth ice/snow* with the geocode wiki of Pangaea (https://wiki.pangaea.de/wiki/Geocode). However, the figure provided in the wiki does not clearly enough define reference depth.

### Step 4: Technical combination

**Fig. 4:**
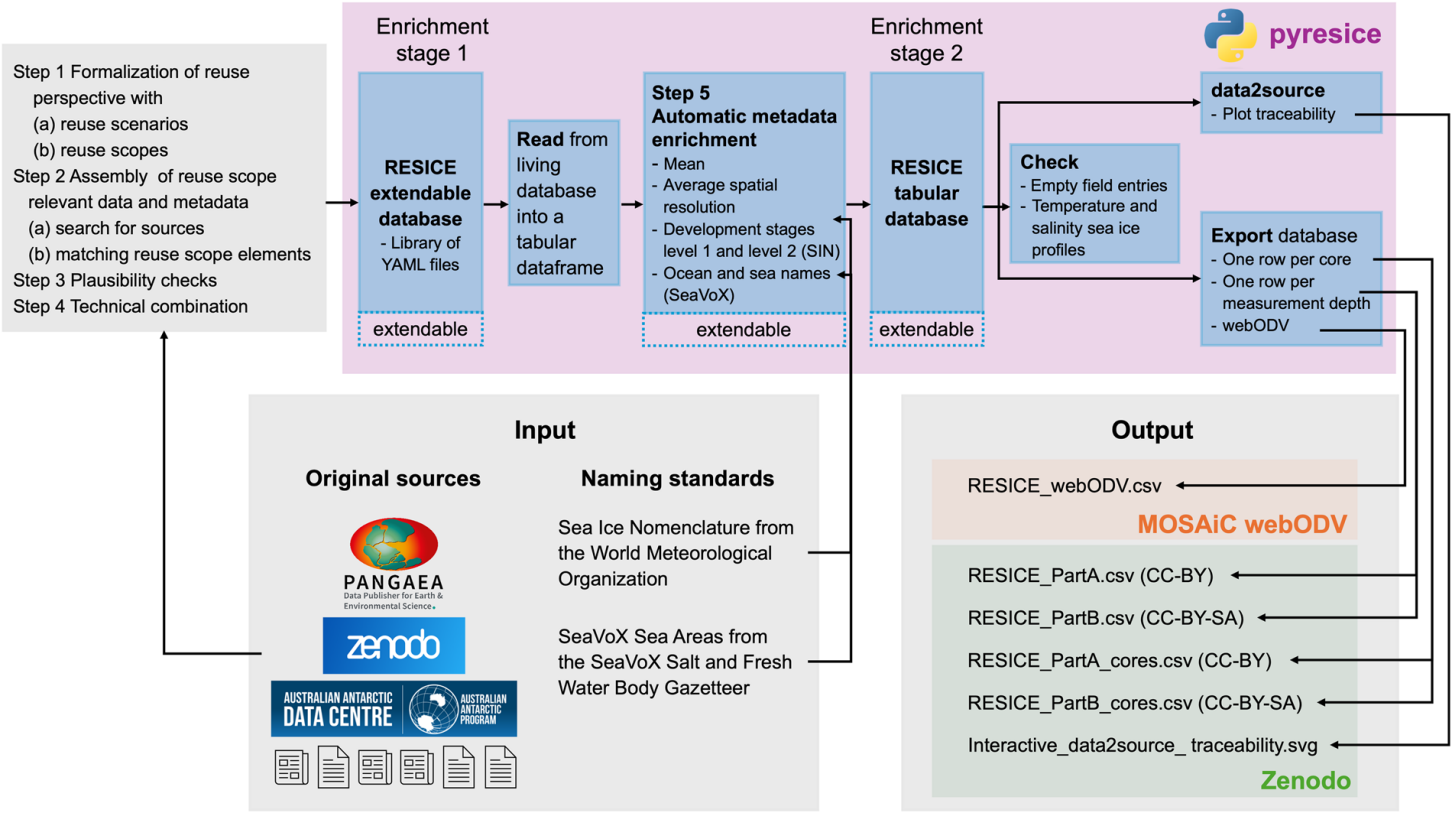
Shows the RESICE tabular database preparation steps. Starting from the original sources, the RESICE extendable database is created following Steps 1 to 4. The YAML files of the extendable database are then merged into a tabular dataframe, which is automatically enriched in Step 5. The RESICE tabular database is then checked for empty field entries and consistency of the measurement profiles. Next, the output files (lower right box) are exported. They are available in Zenodo and the MOSAiC webODV. Each blue box in the upper purple box indicates a module of the pyresice Python package. RESICE extendable and tabular databases can be extended either by adding new YAML files to the RESICE extendable database, new fields to existing YAML files, or new routines to the automatic_enrichment module. Lastly, the data2source module contains the function to create the plot Interactive_data2source_traceability.svg file.

In the next step, the selected sources are technically combined. The availability matching follows a logic that is individual per core and cannot easily be automated (if at all). Therefore, each core should be represented in a separate file instance. This file should allow reproducibility of the matching process and flexibly store elements of different nature, e.g., strings, tabulated data, scalars. We choose the YAML format due to its flexibility. The elements matched from sources have to be made available in Python and then written into YAML files. We refer to the ensemble of YAML files as RESICE extendable database. It is *extendable* since it is hosted in a public GitLab repository, and, therefore, allows for pull requests and issue reporting from the community in the future. The instantiated YAML files are all set up in the same scheme, so they can be subsequently combined into the RESICE tabular database by merging the YAML files into a tabular data frame in Python. Figure 4 highlights the major differences between the extendable and tabular databases. RESICE extendable database is the result of Steps 1 to 4 and only contains data and metadata from existing sources, while the RESICE tabular database also contains automatically enriched metadata as will be explained in Step 5. Thus, the databases represent two different enrichment stages of RESICE, namely enrichment stages 1 and 2, as annotated in Fig. 4. The RESICE tabular database constitutes the final product of the compilation process. We refer to elements in context of reuse scope, fields in context of YAML files and columns in context of the tabular database. In the following, the creation of the YAML files is explained.

#### Supplying sources to Python

The process of loading primary sources into Python for the initiation of the YAML file writing process depends on the data set formats and structures as they are provided on the data repositories. Data sets from Pangaea can be loaded directly into Python as Pandas DataFrames using the panageapy package^64^. The data files from Zenodo and AADC data sets are downloadable via API, for instance, with the datahugger Python package (https://github.com/J535D165/datahugger). However, the downloaded data files are not readily available in Python. The data files first have to be read in a next step. Another option is to download the data files directly from the data repositories.

Some data files from Zenodo and AADC are not efficiently machine readable and have to be manually adjusted before they can be loaded into a Pandas dataframe. Examples are the data sets from Meiners^40^ and Wang *et al*.^22^, where the data files are structured in an unsystematic form with repeated column names for each core. Once the data is supplied to Python, it is written to YAML files. Different scripts for YAML file writing are required depending on the content and naming scheme of the individual data sets. Elements matched from secondary and tertiary sources are manually provided to the script.

#### Harmonization

Matched elements have to be harmonized, when transferred to the YAML files. Harmonization includes label names, classes, units, coordinates, dates and measurement depths.

- Label names used for the same elements vary between sources, but they have to be consistent across all YAML files. Therefore, a naming convention for the YAML files is necessary. We choose an underscore as the join between the property name (e.g., date, temperature, thickness) and the material (e.g., sea ice, air) such as *temperature_air*. Properties or material names that consist of several words such as *measurement-device-accuracy-temperature_sea-ice* are separated with a hyphen. Column labeling of the tabular database follows the same scheme with the only difference that hyphens are replaced by underscores. For reference, a list of all YAML file field names and tabular database column labels is provided in the Data Records.
- For categorical elements such as *form_sea-ice* and *development-stage_sea-ice*, there exist different naming for the same classes, which requires name harmonization. Examples are *multi-year ice* and *multiyear ice* as well as *landfast ice*^37^ and *fast ice*. We adapt the respective class names to SIN classes if they are consistent.
- Units and ratios need to be consistent across all YAML files. Temperature in Celsius (*T*_*C*_) has to be converted to temperature in Kelvin (*T*_*K*_) following *T*_*K*_ = *T*_*C*_  +  273.15. If the unconverted *T*_*C*_ temperature has only one decimal place, the converted temperature in *K* will be rounded accordingly. Salinity is in principle unit less, but it is assigned a ratio. The data is given in either practical salinity unit (psu) or parts per thousand (ppt), which is equivalent to *g* *kg*^−1^. Salinity is converted from psu (*S*_*psu*_) to ppt (*S*_*ppt*_), using the formula ${S}_{ppt}={S}_{psu}\frac{35.16504}{35}\,g\,k{g}^{-1}$ from Millero *et al*.^65^, which may affect changes of the second decimal place.
- Coordinates must be converted if they are not in decimal degrees. This is the case for the data sets from Audh *et al*.^23^ and Meiners^40^, who use degrees, minutes, and seconds.
- The date format has to be converted if it does not follow YYYY-MM-DD, such as the data set from Meiners^40^.
- Temperature is measured point wise at locations along the core, and salinity is measured volume wise per melted core sections. For salinity measurements, the measurement depth has to be adjusted if two depths, i.e., the bottom and the top of a section, are assigned to one measurement. The center of the section is calculated and used as depth.

#### YAML file format and scheme

The elements matched from the sources are combined in YAML files with one file instance per sea ice core. Each YAML file follows a structure of fields and sub-fields as exemplified in Table 6. The assignment of reuse scope elements to YAML file fields follows the reuse scope, unless a hierarchical combination of elements within one field and its sub-fields is logical. For instance, instrument accuracy of a thermometer would be hierarchically a sub-field of the *temperature_sea-ice* field, and standard deviation of sea ice thickness would be a sub-field of the *thickness_sea-ice* field. However, instrument accuracy usually comes from a different source than the temperature data. Thus, it is represented as a separate field, while the relationship of the two fields is obvious from the naming convention. On the contrary, for data set by Wang *et al*.^22^ standard deviation for sea ice thickness is provided in the same source as the measurement data. Thus, standard deviation is added as sub-field to the *thickness_sea-ice* field.

**Table 6:** General description of YAML file sub-fields (a) with examples (b) for the fields *temperature_sea-ice* and *thickness_sea-ice*.

General (a)		Examples (b)	
Sub-field	Description	temperature_sea-ice	thickness_sea-ice
type*	Defines the type of the element as string/scalar/tabulated	tabulated	scalar
value*	Text/float/dictionary	{0.05: 271.50, 0.15: 271.25, 0.25: 270.70, [...]}	0.825
unit_str	Standard unit symbol for the value	K	m
unit	Unit of the value in machine readable format [kg m s K A mol cd]	[0 0 0 1 0 0 0]	[0 1 0 0 0 0 0]
comment	Matching and plausibility - relevant excerpt, table or figure - inconsistencies or adjustments	from C to K	Sea ice thickness is not explicitly available. Substituted by depth of lowest measurement.
variable	Labels for the dictionary keys if type is tabulated	depth ice/snow	—
variable_unit_str	Standard symbols for the units of the keys if type is tabulated	m	—
variable_unit	Unit of the dictionary key if type is tabulated in machine readable format [kg m s K A mol cd]	[0 1 0 0 0 0 0]	—
source*	Abbrevation of the source as listed in Table 3	Torstensson_*et_al*_2018a	Audh_*et_al*_2022
doi*	doi or url of the source	10.1594/Pangaea.924295	10.5281/zenodo.6997630
adjusted	Indicates adjustment of temperature and salinity measurement data with respect to the original source adjusted: 1.0, equivalent to original: 0.0	1.0	—

The sub-fields marked with an asterisk are compulsory for every field. Other sub-fields are required depending on the field’s type and the peculiarities of the availability matching and harmonization process, which are documented in the sub-field *comment*. The sub-fields involving *variable* are only required for elements of type tabular, and the sub-field *adjusted* is only relevant for numerical measurement data, namely temperature and salinity.

The general structure of the fields and sub-fields together and with examples for *temperature_sea-ice* and *thickness_sea-ice* are provided in Table 6. Each field’s type is defined by sub-field *type*, which can be coordinate, string, scalar, or tabulated. The type defines the form of the sub-field *value*. If type is tabulated, such as *temperature_sea-ice*, sub-field *value* is of form dictionary with each key representing the measurement depth and each value representing a measurement. The sub-fields *unit_str* and *unit* define the unit of *value*, where the former is a human-readable version and the latter defines the unit with systematically documented base SI-Units so that [0 0 0 1 0 0 0] represents the unit Kelvin according to [kg m s K A mol cd]. The same holds for the units of *variable*, which defines the unit of the keys for *type* tabulated. In case of *temperature_sea-ice*, it would be *depth ice/snow*. Each field has a single source. The source of each field is provided with its name as sub-field *source* and its doi as sub-field *doi*. If no doi is available, it is filled with the url.

#### Traceability

RESICE requires reproducibility of the availability matching and potential changes with respect to their original sources due to plausibility checks or harmonization. We implement this in RESICE through several traceability options. First, each YAML file field has sub-fields *source* and *doi*, where the name and doi/url of the original sources are stored. These fields allow users to reach the original sources quickly. For elements matched with direct hits and direct availability, these fields are sufficient to ensure the process is comprehensible and reproducible. As soon as elements are matched through indirect hits or indirect availability or their units need to be harmonized, more options for a traceable documentation are required. Examples are elements that were matched from a table of a secondary source, which has to be documented, or subjective decisions in the matching process, such as when missing sea ice thickness is matched with the depth of the lowest measurement. For these cases, we use the sub-field *comment* in the YAML file, where the process is commented (e.g., table number, excerpt citation). The *comment* sub-field is also used to comment changes due to harmonization and plausibility checks.

A second traceability option is required to transparently document the inconsistent provision of the same element from different sources, for instance, two different water bodies or sea ice development stages. In this case, an extra field is added to the YAML file, which is named after the actual field name combined with the suffix *option*. The field without the suffix is the field that will be transferred to the final RESICE tabular database. Table 7 shows an example of the traceability options for the field *development-stage_sea-ice*. The element can be matched from two sources that provide conflicting classes for sea ice development stages. Therefore, both development stages are stored in the YAML file with separate fields, one with suffix *option*. Additionally, the sub-fields *comment* provide details on the excerpt and the table from where the fields were matched. The sub-field *adjusted* is added to highlight changes of the measurement data as saved in the YAML file with respect to the original sources. It is only provided for temperature and salinity measurements, and it is 1.0 if changes such as unit conversion and plausibility checks have been conducted. In all other cases, it is equivalent with the original source and filled with 0.0.

**Table 7:** Example for the reproducibility of the matching process for the element development stage sea ice represented as YAML file field *development-stage_sea-ice* of sea ice core with ID *PS69_584-1_WS-21*.

Sub-field	development-stage_sea-ice	development-stage_sea-ice_option
type	string	string
value	multi-year ice	first-year ice
comment	The samples from stations WS-4, WS-7, WS-11, and WS-21 were multi-year ice covered with second-year snow, whereas the samples from all other stations were first-year ice (Haas *et al*., 2009, Willmes *et al*., in press).	from Table 1
source	Kramer_*et_al*_2011	Lemke_2009
doi	10.1016/j.dsr2.2010.10.029	10.2312/BzPM_0586_2009

The development stage for this sea ice core is provided inconsistently from two sources. Therefore, the field *development-stage_sea-ice_option* had to be added to the YAML file of this sea ice core. For the field *development-stage_sea-ice*, the sub-field *comment* contains an excerpt from a secondary source, which defines the development stage, and for the field *development-stage_sea-ice_option*, the *comment* sub-field documents the table of a secondary source from where the development stage was matched.

#### Creation of the tabular database

The RESICE tabular database is created by reading in the YAML file fields including the sub-fields *value*, *source*, *doi*, and if available *comment* and *adjusted*, into a Pandas dataframe in Python. Each sub-field is transferred to a column with a name that combines YAML file field and sub-field names (e.g., *temperature_sea_ice_source*). If the field has a sub-field specifying the unit, the unit is combined with the column name (e.g., *temperature_sea_ice [K]*). All hyphens of the YAML file field names are changed to underscores. The fields with suffix *option* are neglected. Instead of combining the measurement depth in tabulated form directly with the measurement values, the tabular database has a *depth* column with all measurement depths that are unique per core. YAML file field *coordinate* is split into *Latitude* and *Longitude* columns in the tabular database. The interested reader can find the functions used to merge the YAML files into the tabular database in the read module of the accompanying Python package pyresice^12^. After the automatic metadata enrichment in Step 5, the tabular database is exported as csv-files as illustrated in the lower right box in Fig. 4.

### Step 5: Automatic metadata enrichment

The YAML files in the RESICE extendable database combine all data and metadata found in primary, secondary, and tertiary sources. Yet, not all elements as requested in the reuse scopes could be matched from sources in Step 2 (b) and are therefore not included in the final RESICE tabular database. In some cases, the data reuser can infer these unavailable elements from already matched elements conserved in the YAML files. For RESICE, we propose Python routines to systematically derive missing elements in an automatic way. It is important to note that the enriched elements are not added to the YAML files. They are directly provided as columns to the RESICE tabular database, which is created by merging the YAML file contents in a tabular dataframe. The workflow is illustrated in Fig. 4, and the automatic enrichment routines are provided in the automatic_enrichment module of the pyresice Python package. The columns *mean_salinity_sea-ice*, *mean_temperature_sea_ice*, *mean_distance_measurements_salinity_sea_ice* and *mean_distance_measurements_temperature_sea-ice*, *sea_SeaVoX*, *ocean_SeaVoX*, *development_stage_SIN_level_1_sea_ice* and *development_stage_SIN_level_2_sea_ice* are automatically enriched as described in the following.

#### Mean distance of the measurements from temperature and salinity sea ice

To enable Reuse Scenario A, the mean distance of the measurements of the temperature and salinity sea ice along the core are computed. The distances are calculated and then averaged for all profile measurements. The mean distance of the measurements is automatically enriched for all sea ice cores that provide profile measurements for temperature and salinity.

#### Mean values of temperature and salinity sea ice

To enable Reuse Scenario B, the mean value per core is calculated from the profile measurements of sea ice temperature and salinity by averaging all measurement values. The mean value of the measurement data is automatically enriched for all sea ice cores that provide the respective temperature and salinity measurements.

#### Development stage sea ice form SIN level 1 and level 2

To enable Reuse Scenario B, development stage of sea ice has to follow the classes of the Sea Ice Nomenclature (SIN)^15^ in a consistent way. However, the development stages for sea ice provided in the sources do not always match a SIN class, or they are used in a cross-categorical manner as explained in Step 2 (b). Therefore, two columns are automatically enriched in the RESICE tabular database. They are *development-stage-SIN-level-1_sea-ice*, which refers to all classes on the level 1, and *development-stage-SIN-level-2_sea-ice*, which refers to all classes on the level 2 of the SIN. More specifically, *development-stage-SIN-level-1_sea-ice* and *development-stage-SIN-level-2_sea-ice* are derived based on the YAML file fields *development-stage_sea-ice* and/or *thickness_sea-ice*, which were matched from sources as explained in Step 2 (b). We use a dictionary of all SIN class names of level 1 and 2 to derive the level of the class of *development-stage_sea-ice*. If it corresponds to level 1, subordinate level 2 is derived using *thickness_sea-ice* by matching it with the characteristic thickness ranges of the level 2 classes as listed in Table 2. If it corresponds to level 2, superordinate level 1 is easily derived as it is the parent of level 2. It may occur that the YAML file field *development-stage_sea-ice* is consistent with a level 1 or level 2 class from the SIN, while the YAML file field *thickness_sea-ice* does not match the corresponding characteristic thicknesses of the respective SIN class. In this case the YAML file field *development-stage_sea-ice* is neglected, and *development-stage-SIN-level-1_sea-ice* and *development-stage-SIN-level-2_sea-ice* are derived based on the *thickness_sea-ice* alone, i.e., the corresponding level 2 class is matched based on the thickness following the thickness ranges in Table 2 and then superordinate level 1 is derived. The same applies to the case when *development-stage_sea-ice* is not available from a source or it does not match with a SIN class. In these cases, we issue a warning that is stored in the RESICE tabular database column *INFO_SIN*. The reader should note that the SIN classes corresponding to new ice, pancake ice, and ice rind are excluded from the automatic enrichment routine because they are not associated with sea ice thicknesses or intersect with other characteristic thicknesses. Furthermore, the characteristic thickness of the level 2 class *residual ice* intersects that of *first-year ice*. To avoid conflicts of class assignment with the automatic enrichment routine, *development-stage-SIN-level-2_sea-ice* can only be *residual ice* if the YAML file field *development-stage_sea-ice* is equal to *old ice* or *residual ice*. If the automatic enrichment is based only on the *thickness_sea-ice* field due to missing details on the development stage from sources, it will only assign classes of *first-year ice* even if it could also be residual ice. In this case the result should be treated with caution. A warning is issued in the column *INFO_residual_ice*.

#### Sea and ocean from SeaVoX

The names for water bodies as they are provided in the sources and stored in the YAML file field *water-body* do not comply with a controlled vocabulary. Liza’s Reuse Scenario B requires names defined with the *SeaVoX Salt and Fresh Water Body Gazetteer*^16^. Therefore, we automatically enriched the two elements *ocean-SeaVoX* and *sea-SeaVoX*, which are assigned the SeaVoX attributes *OCEAN* and *SUB_REGION*. More specifically, we loaded the *Polygon data set of the extent of water bodies* shapefile into Python using GeoPandas and then evaluated each core’s coordinates as stored in the YAML files for the polygon attributes *REGION*, which is equivalent to *ocean-SeaVoX*, and *SUB_REGION*, which is equivalent to *sea-SeaVoX*.

## Data Records

The static version of the RESICE tabular database is available in its newest version in csv-format from Zenodo^10,11^. The full RESICE tabular database has the length of the amount of unique measurement depths of the sea ice salinity and temperature measurements of all cores. It includes 2745 sea ice temperature measurements and 2862 sea ice salinity measurements. In total, it has 4327 rows since many salinity and temperature measurements of the core are assigned the same depth.

As different licenses apply to the original sources of the data, the RESICE tabular database is split into two Zenodo entries. The data sets are provided in RESICE - Reusability Enhanced Sea Ice Core Database - Part A^10^ with CC-BY license and Part B^11^ with CC-BY-SA license. Part A and Part B of the database are linked via RESICE - Reusability Enhanced Sea Ice Core Database - General Information^66^ providing metadata and information on the column labels. The Zenodo entries of Part A and Part B contain the following csv-files.

- RESICE_PartA.csv/RESICE_PartB.csv: These csv-files contain the database with one row per measurement depth of the sea ice salinity and temperature measurements. The column labels of the csv-files are listed in the Table 8.
- RESICE_PartA_cores.csv/RESICE_PartB_cores.csv: These csv-files constitute a reduced version of the RESICE tabular database. In this case, the columns representing the profile measurement data, i.e., *depth*, *salinity_sea_ice* and *temperature_sea_ice* are neglected. Part A and B combined have 287 rows so that one row represents one sea ice core.
- sources.csv: This csv-file lists the respective sources, licenses and dois/urls used to create the csv-files.

Additionally to the tabular database, we also provide the RESICE extendable database, i.e., the ensemble of YAML files, in the pyresice Python package available via GitLab^12^. Specifically it is provided in module RESICE_extendable_database in the folder src/pyresice. The YAML file field names are listed and explained in Table 8 together with the column labels of the tabular database.

**Table 8:** Description of the YAML file field names and tabular (Tab.) database column labels.

Field name/Column label	Unit	Description	YAML	Tab.
depth	m	depth value of the sea ice temperature and salinity measurements		*✓*
development-stage-SIN... -level-1_sea-ice and -level-2_sea-ice	m	term as classified in Development Section 2 of SIN^15^ sub-categories level 1 (2.x) and level 2 (2.x.y)		✓*
INFO_residual_ice		info on intersection with residual ice		✓*
INFO_SIN		info on SIN automatic enrichment		✓*
latitude		in decimal degrees		*✓*
latitude		in decimal degrees		*✓*
longitude		in decimal degrees		*✓*
mean-distance-measurements... -salinity_sea-ice and -temperature_sea-ice	m	mean distance between measurements of one core salinity sea ice and temperature sea ice		✓*
mean... -salinity_sea-ice and -temperature_sea-ice	ppt K	mean values of all measurements of salinity of sea ice and temperature of sea ice		✓*
ocean-SeaVox		ocean from SeaVoX^16^ derived from coordinates		✓*
sea-SeaVox		sea from SeaVoX^16^ derived from coordinates		✓*
[*column label*]_adjusted		indicates adjustment of values w.r.t. original sources		*✓*
[*column label*]_comment		excerpt/figure/table/inconsistencies/adjustments		*✓*
[*column label*]_doi		doi (sometimes url) of the source		*✓*
[*column label*]_source		name of the source as listed in Table 3		*✓*
calculation-method-temperature_sea-ice		potential computational manipulations of the measurement data (e.g., interpolation)	*✓*	*✓*
campaign		name of campaign/expedition/project	*✓*	*✓*
date		date of core retrieval, in YYYY-MM-DD format	*✓*	*✓*
development-stage_sea-ice		sea ice age classification	*✓*	*✓*
form_sea-ice		pack ice, drift ice, or fast ice as called in the source	*✓*	*✓*
freeboard_sea-ice	m	sea ice thickness above water level	*✓*	*✓*
id		combination of campaign name and core number	*✓*	*✓*
measurement-device-accuracy... -salinity_sea-ice, -temperature_air and -temperature_sea-ice	ppt K K	accuracy of measurement device salinity sea ice, temperature air and temperature sea ice	*✓*	*✓*
measurement-device... -salinity_sea-ice, -temperature_air and -temperature_sea-ice		name of measurement device for salinity sea ice, temperature air and temperature sea ice	*✓*	*✓*
polar-region		Arctic or Antarctica	*✓*	*✓*
salinity_sea-ice	ppt	bulk salinity of sea ice (in YAML combined with depth)	*✓*	*✓*
temperature_air	K	temperature of air	*✓*	*✓*
temperature_sea-ice	K	temperature of sea ice (in YAML combined with depth)	*✓*	*✓*
thickness_sea-ice	m	thickness of sea ice	*✓*	*✓*
thickness_snow	m	thickness of snow cover on top of sea ice	*✓*	*✓*
water-body		name of water body at the coring location	*✓*	*✓*
name		name of the core in the extendable database	*✓*	
coordinates		"N”: 74.70933, “E”: -95.24408, in decimal degrees	*✓*	
[*field name*]_option		in case two sources provide the same field (see Table 7)	*✓*	

Column labels of the tabular database assigned with an asterisk alongside the check mark are automatically enriched as described in Step 5. Coordinates in the YAML files are transformed into Latitude and Longitude columns in the tabular database. For profile measurements temperature and salinity sea ice, depth is combined with the measurements in the YAML files. In the tabular database, depth is a separate column. The column labels starting with *[column label]* represent the YAML file sub-fields as listed in Table 7 in the tabular database. Placeholder *[column label]* is then replaced by any other column label such as *salinity_sea-ice*.

## Technical Validation

RESICE reuses existing data. Therefore, the goal of the technical validation is not to prove the quality of the measurement data. Instead, the consistent transfer of the data from the original sources to RESICE has to be ensured and tested. Additionally, the described reproducibility of the database enrichment process and the traceability of the data points back to their original sources has to be demonstrated.

### Consistency of RESICE

The consistency of the data points transferred to RESICE with respect to the original sources has to be validated. We performed a random draw of four fields, each representing measurement data (e.g., salinity, temperature) from different YAML files. All *adjusted* subfields of the selected fields must be 0.0, which indicates they were not adjusted in the database preparation process and should be equivalent to the original sources. The values of these four selected fields were compared with the data provided in the original sources. The YAML file data was consistent with the original data.

**Fig. 5:**
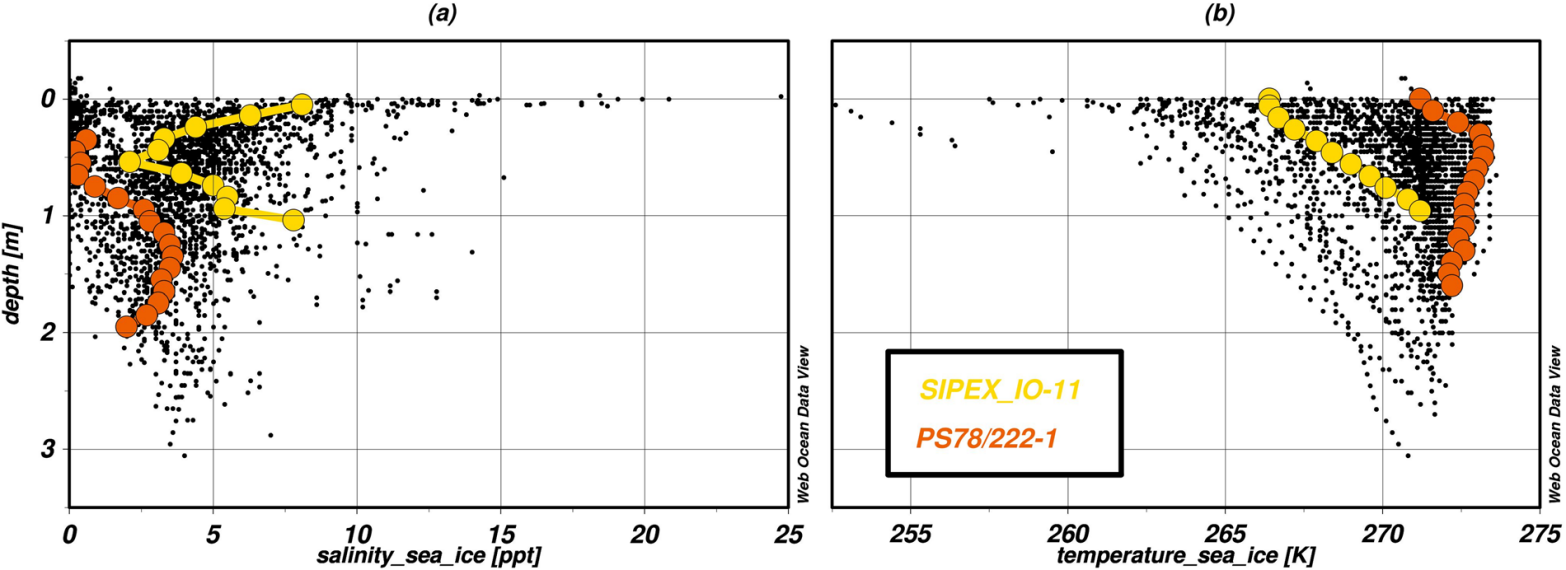
All sea ice temperature (**a**) and salinity (**b**) measurements of the RESICE tabular database combined in a scatter plot. The profiles for the two sea ice cores with IDs *SIPEX_IO-11* and *PS78/222-1* are highlighted. This figure was prepared with webODV and is available in an interactive version (https://mvre.webodv.cloud.awi.de/DataExploration/id/DVevtE7c/sal_temp_scatter).

Additionally, the consistency of the combined database is demonstrated with the scatter plot of all sea ice salinity and temperature measurements in Fig. 5. The temperature values are in the physical range between 250 K and 274 K and the salinity values between 0 and 25 ppt. All measurement data is assigned a depth value. In Fig. 5, measurements with negative depth are measurements in the snow, and the deepest measurement depth is 3.1 m. Further figures that underline the physical consistency of RESICE are available in the MOSAiC webODV. We provide figures that combine plots of the evolution of sea ice thickness over time, the mean sea ice salinity with respect to sea ice thickness and sea ice freeboard, as well as sea ice mean temperature with respect to thickness of snow cover for the Arctic (https://mvre.webodv.cloud.awi.de/DataExploration/id/DVevtE7c/arctic_correlation) and the Antarctic (https://mvre.webodv.cloud.awi.de/DataExploration/id/DVevtE7c/antarctic_correlation).

### Traceability of the original data sources

The checkerboard in Fig. 3(a) provides an overview of the available fields per YAML file of the RESICE extendable database and the colors indicate the type of source group the fields are matched from. The plot is ordered vertically by campaign, so that cores from the same campaign are listed one below the other. All YAML files are indexed on the y-axis. So that each row refers to one YAML file (equivalent to one sea ice core). The YAML file field names are listed on the x-axis.

Some fields are available for all YAML files. They are *date*, *coordinates*, *campaign*, *polar-region*, *water-body*, *form_sea-ice*, and *thickness_sea-ice*. All other fields are not always available. Fields that are majorly unavailable are *calculation-method-temperature_sea-ice* and those related to *temperature_air*. For example, for sea ice core with index 0, several fields are available from a primary source. The development stage of the sea ice and the names of the measurement devices for salinity and temperature are available from a secondary source. A secondary source furthermore provides information on the calculation method for temperature sea ice. The accuracies of all measurement devices and measurement data for air temperature and sea ice freeboard are not available.

The line plot Fig. 3(b) emphasizes the traceability of each data point in RESICE. Gray nodes each represent one YAML file (sea ice core). The indexing of the gray nodes is equivalent with that of the the y-axis of the checkerboard in Fig. 3(a). Green, orange, and pink nodes each represent individual primary, secondary, and tertiary sources, respectively. Accordingly, each colorful dot represents one doi/url used in RESICE. The plot illustrates the distribution of sources combined per YAML file. The lines between primary source nodes and secondary source nodes show that each YAML file’s fields are filled from one or more primary sources and one or more secondary sources. Furthermore, some YAML files have fields, which are filled from tertiary sources. As tertiary sources are unrelated to the cores, the same tertiary node can be connected with YAML files from different campaigns. We provide Fig. 3(b) as an interactive image file in svg-format in Zenodo^67^. In this interactive file, each colorful node is directly linked to the respective doi/url of the original source, and each gray node links to the respective YAML file in the RESICE extendable database available in the pyresice package on GitLab. The reader should not that the lines plot includes all sources considered for the YAML file generation, also those used for fields with suffix *option*.

## Usage Notes

The main goal of RESICE is to enable reuse scenarios that require sea ice core data by lowering the data access threshold. Therefore, we facilitate the use of RESICE by providing it on three different platforms. 1) RESICE tabular database is interactively viewable and analyzable on the web in the MOSAiC webODV. 2) RESICE tabular database is published in csv-format in Zenodo. 3) RESICE extendable database is provided in the pyresice Python package in GitLab, which includes all routines required to generate the RESICE tabular database. In this Usage Notes, we provide further information to support the use of RESICE.

### Interactive webtool: MOSAiC webODV

RESICE has been added to the interactive online visualization and analysis tool MOSAiC webODV, where it is available as supporting data for analysis of data from the MOSAiC campaign. We provide several figures that can be accessed via the collection (https://mvre.webodv.cloud.awi.de/DataExploration/id/DVevtE7c). The reader should note that before accessing RESICE, a webODV login is necessary. Anonymous login is possible. After reaching the RESICE collection, figures can be selected via *view* on the top right and then via *load views*. Additionally to the scatter plot in Fig. 5, correlation plots for Antarctica (https://mvre.webodv.cloud.awi.de/DataExploration/id/DVevtE7c/antarctic_correlation) and the Arctic https://mvre.webodv.cloud.awi.de/DataExploration/id/DVevtE7c/arctic_correlationas well as a plot for the development stages (https://mvre.webodv.cloud.awi.de/DataExploration/id/DVevtE7c/dev_stage) are available. The user can furthermore generate custom plots. The csv-file used to integrate RESICE in MOSAiC webODV can be generated with the respective function of the export module in pyresice.

### Tabular database: RESICE on Zenodo

If users plan to use RESICE in their work, we suggest to download the tabular database from Zenodo. As explained in the Data Records, we added two Zenodo entries for the RESICE database due to the different licenses of the original data sources. Therefore, we suggest to load the csv-files of Part A and Part B into Python by creating a Pandas DataFrame, merge the data frames, and reduce the database to the columns required for the specific scenario. If the RESICE extendable database is changed, a new version of the tabular database will be provided via Zenodo.

It should be noted that all column labels with suffixes *doi*, *source* and *comment* provide metadata on the origin of the respective data point and are equivalent to the content of the respective YAML file sub-fields. When working with the files RESICE_PartA.csv/RESICE_PartB.csv users should note that negative depths in the *depth* columns indicate that *temperature_sea-ice* and *salinity_sea-ice* were measured in the snow.

### RESICE extendable database and automatic enrichment: pyresice Python package

We suggest to use pyresice for extension of RESICE or reproducing the output data files and interactive data2source traceability plot. The Python package is available via GitLab. It currently is a local Python package created based on the template provided by Cookiecutter. The package can then be imported using Poetry (https://github.com/python-poetry/poetry). The package is licensed with GPLv3 since the RESICE extendable database also contains data licensed by CC-BY-SA. The general package structure is illustrated in the purple box on the top right in Fig. 4. Each blue box represents a module of the package, i.e., a sub-folder in the src/pyresice folder of the package.

RESICE can be extended either via the module 1) RESICE_extendable_database or 2) automatic_enrichment.

1. The RESICE extendable database can be extended by adding new parameters to a sea ice core, i.e., adding new fields to existing YAML files. For instance, snow thickness for sea ice core with ID *PS78/230-1* is missing. A user, who knows a source that provides the snow thickness for this core, could add a *thickness_snow* field in the respective YAML file. When adding a new field, the YAML structure as explained in Table 6 and the naming scheme as explained in the readme file (https://git.rwth-aachen.de/mbd/pyresice/-/blob/main/src/pyresice/RESICE_extendable_database/yaml_db/readme.md) of the module RESICE_extendable_database should be followed. Furthermore, the RESICE extendable database can be extended by instantiating a new YAML file and adding data and metadata of a new sea ice core. For instance, if users have been part of a field campaign and have measured salinity of sea ice cores that are not yet part of RESICE, they could add a new YAML file for each new core to the database. For the extension of RESICE with additional sea ice cores the procedure is the same as described in the Methods. The original sources have to be checked for plausibility, supplied to Python, harmonized and then combined in a YAML file. The reader should note that a YAML file can also be generated manually and without a Python script.
2. The RESICE tabular database can be extended by adding functions to the automatic_enrichment module. There may exist another scheme for the classification of the development stages of sea ice that is of interest for a data reuser. This scheme could be transferred into a Python routine and then added to the module.

Generally, extensions can be added to RESICE through a pull request via the GitLab repository. The pull request would be granted after a quality check of the new data such as compliance with the standard format.

If users add new YAML files, they should note that the automatic enrichment routine enrich_seaVoX for the elements *sea-SeaVoX* and *ocean-SeaVoX* is very time intensive. Therefore, we provide a mapping for all coordinates that are currently part of the database. If users add a new sea ice core with new coordinates to the extendable database, this mapping has to be extended by running first the check_for_new_coordinates, second the map_new_coordinates and third the extend_mappings functions. The functions are all part of the automatic_enrichment module.

Pyresice furthermore contains a check module, which contains the function overview_10 to create a matrix plot for all rows and columns of the database. The entries of the matrix are either zero or one, depending on whether the entry is empty or not. If one of the columns is zero for all cores, there may be a spelling mistake in the code. The check module also includes the function *plot_temp_salinity_combined*, which creates an interactive plot that allows users to scroll through the salinity and temperature profiles of all sea ice cores.

The export module creates two different versions of the RESICE tabular database. One is the full database (RESICE_PartA.csv and RESICE_PartB.csv) and the other one is the reduced version without profile measurements (RESICE_PartA_cores.csv and RESICE_PartB_cores.csv). Additionally, the export module creates a version of RESICE for the integration in MOSAiC webODV. Lastly, the data2source module provides the functions used to generate the interactive data2source traceability plot in Fig. 3.

## Data Availability

The code used for this study is available in the pyresice Python package on Zenodo^12^. Version v0.1.1 is the subject of this article.
